# Genetic and physical mapping of the earliness *per se* locus *Eps-A*^*m*^*1* in *Triticum monococcum* identifies *EARLY FLOWERING 3* (*ELF3*) as a candidate gene

**DOI:** 10.1007/s10142-016-0490-3

**Published:** 2016-04-16

**Authors:** M. A. Alvarez, G. Tranquilli, S. Lewis, N. Kippes, J. Dubcovsky

**Affiliations:** Department of Plant Sciences, University of California, Mail Stop 1, One Shields Avenue, Davis, CA 95616-8780 USA; Instituto de Recursos Biológicos, INTA, Villa Udaondo (1686), Hurlingham, Buenos Aires Argentina; Howard Hughes Medical Institute, Villa Udaondo (1686), Chevy Chase, MD 20815 USA

**Keywords:** *Triticum monococcum*, Wheat, Earliness *per se*, *ELF3*, Flowering time, Spikelet number

## Abstract

Wheat cultivars exposed to optimal photoperiod and vernalization treatments still exhibit differences in flowering time, referred to as earliness *per se* (*Eps*). We previously identified the *Eps-A*^*m*^*1* locus from *Triticum monococcum* and showed that the allele from cultivated accession DV92 significantly delays heading time and increases the number of spikelets per spike relative to the allele from wild accession G3116. Here, we expanded a high-density genetic and physical map of the *Eps-A*^*m*^*1* region and identified the wheat ortholog of circadian clock regulator *EARLY FLOWERING 3* (*ELF3*) as a candidate gene. No differences in *ELF3* transcript levels were found between near-isogenic lines carrying the DV92 and G3116 *Eps-A*^*m*^*1* alleles, but the encoded ELF3 proteins differed in four amino acids. These differences were associated with altered transcription profiles of *PIF-like*, *PPD1*, and *FT1*, which are known downstream targets of ELF3. Tetraploid wheat lines with combined truncation mutations in the A- and B-genome copies of *ELF3* flowered earlier and had less spikelets per spike than the wild-type control under short- and long-day conditions. Both effects were stronger in a photoperiod-sensitive than in a reduced photoperiod-sensitive background, indicating a significant epistatic interaction between *PPD1* and *ELF3* (*P* < 0.0001). By contrast, the introgression of the *T. monococcum* chromosome segment carrying the *Eps-A*^*m*^*1* allele from DV92 into durum wheat delayed flowering and increased the number of spikelets per spike. Taken together, the above results support the hypothesis that *ELF3* is *Eps-A*^*m*^*1*. The *ELF3* alleles identified here provide additional tools to modulate reproductive development in wheat.

**Electronic supplementary material:**

The online version of this article (doi:10.1007/s10142-016-0490-3) contains supplementary material, which is available to authorized users.

## Introduction

More than 700 million tons of wheat is grown every year in very diverse environments, providing a major source of calories and proteins to the human population. The ability of wheat to adapt to these different conditions has been favored by a rapidly evolving genome combined with the buffering effect of recent polyploidization events (Dubcovsky and Dvorak 2007). This plasticity, coupled with strong selection pressures during the expansion of agriculture and wheat cultivation throughout the world, contributed to a large genetic diversity in genes regulating reproductive development. This diversity has been used by wheat breeders to optimize the utilization of available natural resources during plant growth and grain filling and to maximize grain yield in different environments.

In temperate cereals, such as wheat and barley, the initiation of the reproductive phase is regulated by the integration of two main seasonal signals: photoperiod (day length) and vernalization (prolonged exposure to low temperatures). The photoperiod response is mainly regulated by *PPD1*, a member of the *PSEUDO-RESPONSE REGULATOR* (*PRR*) gene family, which upregulates the expression *FT1* under long days (Turner et al. 2005)*. FT1* encodes a mobile protein, homologous to the Arabidopsis FLOWERING LOCUS T (FT) (Yan et al. 2006), which is transported from the leaves to the shoot apical meristem (SAM) (Corbesier et al. 2007; Tamaki et al. 2007). Once in the apex, FT1 forms a protein complex that binds the promoter of the meristem identity gene *VRN1* (Li et al. 2015) and upregulates its expression above the critical threshold required to initiate the transition from the vegetative to the reproductive stage (Distelfeld et al. 2009). During the fall, the expression of *FT1* in the leaves is downregulated by the flowering repressor *VRN2*, preventing flowering before winter (Yan et al. 2004). During the winter, cold temperatures induce the expression of *VRN1* in the leaves and result in the repression of *VRN2* (Chen and Dubcovsky 2012). In the absence of *VRN2*, the increase of day length during the spring results in the induction of *FT1* and the acceleration of the transition to the reproductive stage (Distelfeld et al. 2009).

Even when the vernalization and photoperiod requirements have been fulfilled, there are still residual differences in flowering time among wheat accessions, which are usually referred to as earliness *per se* (*Eps*) (Flood and Halloran 1984; Hoogendoorn 1985; Worland and Law 1986; Slafer 1996). These differences are affected by temperature (Slafer and Rawson 1994, 1995a, b) and are important for the fine-tuning of flowering and the adaptation of wheat to different environments. Several *Eps* loci have been mapped on different wheat and barley chromosomes (Scarth and Law 1984; Hoogendoorn 1985; Miura and Worland 1994; Laurie et al. 1995; Worland 1996; Bullrich et al. 2002; Griffiths et al. 2009; Gawronski and Schnurbusch 2012; Zikhali et al. 2014, 2015), but only a few of the underlying *Eps* genes have been identified so far (Comadran et al. 2012; Faure et al. 2012; Zakhrabekova et al. 2012; Gawronski et al. 2014).

The temperate cereal homolog of the Arabidopsis circadian clock regulator *LUX ARRHYTHMO* (*LUX*) was proposed as a promising candidate gene for the *Earliness per se 3* (*Eps-A*^*m*^*3*) locus in *Triticum monococcum* L. (2*n* = 14, genomes A^m^A^m^; Mizuno et al. 2012; Gawronski et al. 2014) and for the colinear *early maturity 10* (*eam10*) locus in barely (*Hordeum vulgare* L., 2*n* = 14, genomes HH; Gawronski and Schnurbusch 2012; Campoli et al. 2013). A complete deletion of *LUX* in the *eps-A*^*m*^*3* mutant and a mutation in a highly conserved region of the LUX protein in *eam10* were linked with early flowering under both long-day (LD) and short-day (SD) conditions. The early flowering in both mutants was associated with arrhythmic transcript patterns of central and output circadian clock genes under SD and constant ambient conditions and the upregulation of *PPD1* and *FT1* transcript levels (Mizuno et al. 2012; Campoli et al. 2013; Gawronski et al. 2014). The effects of the *LUX* mutations were larger in the presence of the photoperiod-sensitive allele *Ppd-H1* than in the presence of the photoperiod-insensitive allele *ppd-H1*, suggesting that the effect of LUX was mediated in part by the negative regulation of *Ppd-H1* (Mizuno et al. 2012; Campoli et al. 2013). The *eps-A*^*m*^*3* mutant also showed temperature-dependent variation in spike development (Gawronski et al. 2014).

The *early maturity 8* (*eam8*, also known as *mat-a*) mutants in barley show similar phenotypic effects as the *eam10* mutants described above. The *eam8* gene encodes a homolog of the Arabidopsis circadian clock gene *EARLY FLOWERING 3* (Faure et al. 2012; Zakhrabekova et al. 2012), which is known to interact with LUX and EARLY FLOWERING 4 (ELF4) to form a trimeric protein complex known as the “evening complex” (Nusinow et al. 2011). In Arabidopsis, the evening complex functions as a transcriptional regulator that represses the expression of growth-promoting transcription factors *PHYTOCHROME-INTERACTING FACTOR 4* (*PIF4*) and *PIF5* (Nusinow et al. 2011), as well as *PSEUDO-RESPONSE REGULATOR* (*PRR*) genes *PRR7* and *PRR9* (Dixon et al. 2011; Helfer et al. 2011; Kolmos et al. 2011; Chow et al. 2012; Herrero et al. 2012). The participation of ELF3 and LUX in the same protein complex is consistent with the similar phenotypic effects observed in loss-of-function mutations in either of the two genes, and in their similar epistatic interactions with *PPD1* (Faure et al. 2012; Mizuno et al. 2012; Campoli et al. 2013). Both mutants show early flowering under LD and SD and a transcriptional upregulation of *PPD1*, suggesting that in the temperate cereals the evening complex acts as a repressor of *PPD1* (Faure et al. 2012; Zakhrabekova et al. 2012, Mizuno et al. 2012; Campoli et al. 2013).

In *Triticum aestivum*, a deletion of the chromosomal region including *ELF3* was linked to the earliness *per se* locus *Eps-D1*, which causes early flowering and altered expression of circadian clock gene *GIGANTEA* (*GI*) (Zikhali et al. 2015). The earliness *per se* gene *Eps-A*^*m*^*1* from *T. monococcum* was mapped on the distal region of chromosome arm 1A^m^L, which is colinear to both *eam8* and *Eps-D1* (Bullrich et al. 2002; Valarik et al. 2006; Zakhrabekova et al. 2012). The *Eps-A*^*m*^*1* allele from cultivated *T. monococcum* ssp. *monococcum* (accession DV92) was associated with delayed heading time and increased number of spikelets per spike relative to the allele from wild *T. monococcum* ssp. *aegilopoides* (accession G3116). The effect of this locus was larger when plants were grown at 16 °C compared to 23 °C, suggesting a role of temperature on the modulation of the effects of this gene (Bullrich et al. 2002; Appendino and Slafer 2003; Lewis et al. 2008).

In this study, we developed a high-density genetic and physical map of the *Eps-A*^*m*^*1* region and show that *ELF3* is a strong candidate gene for *Eps-A*^*m*^*1*. We found four amino acid changes between *Eps-A*^*m*^*1* alleles that were associated with differences in the transcription profiles of circadian clock genes and *ELF3* downstream targets. Tetraploid wheat lines carrying loss-of-function mutations in both the A- and B-genome copies of *elf3* showed early flowering and reduced spikelet number and exhibited significant epistatic interactions with *PPD1* for both traits under SD and LD conditions. By contrast, introgression of the *Eps-A*^*m*^*1* allele from cultivated *T. monococcum* accession DV92 into durum wheat significantly delayed flowering time and increased the number of spikelets per spike.

## Materials and methods

### Materials

We developed diploid wheat near-isogenic lines (NILs) for the *Eps-A*^*m*^*1* alleles by backcrossing six times the *Eps-A*^*m*^*1* locus from the wild *T. monococcum* ssp. *aegilopoides* accession G3116 (winter growth habit) into the cultivated *T. monococcum* ssp. *monococcum* accession DV92 (spring growth habit). The *Eps-A*^*m*^*1* allele from G3116 is associated with early heading and is referred to as *Eps-A*^*m*^*1-e*, whereas the allele from DV92 is associated with late heading and is referred to as *Eps-A*^*m*^*1-l* (Lewis et al. 2008). After six backcrosses to DV92, these NILs are expected to be more than 98 % identical to the recurrent parent. We confirmed that both NILs have a spring growth habit determined by a nonfunctional *vrn-A*^*m*^*2* allele (Yan et al. 2004) and that both are photoperiod sensitive (early flowering under LD and extremely late flowering under SD) (Appendino and Slafer 2003).

The tetraploid wheat cultivar Kronos (*Triticum turgidum* ssp. *durum*, 2*n* = 28, genomes AABB) was used to generate the TILLING population analyzed in this study (Uauy et al. 2009). This population included 1536 EMS-mutagenized Kronos lines, with DNAs organized in 384 pools of four individual DNAs (Uauy et al. 2009). Kronos carries the *Vrn-A1c* allele for spring growth habit (Fu et al. 2005; Chen and Dubcovsky 2012) and the *Ppd-A1a* allele for reduced photoperiodic response (Chen et al. 2014). Plants carrying the *Ppd-A1a* allele are usually referred in the wheat literature as “photoperiod insensitive” because they flower earlier under SD than plants carrying the wild-type *Ppd-A1b* allele, reducing the differences in flowering between LD and SD. However, we prefer the term “reduced photoperiodic response” because plants carrying the *Ppd-A1a* allele still flower significantly earlier when grown under LD than when grown under SD, demonstrating that this allele is still able to respond to differences in photoperiod (Chen et al. 2014).

### High-density genetic map of *Eps-A*^*m*^*1*

Seeds for the high-density map were generated from BC_6_ and BC_6_F_2_*T. monococcum* NILs heterozygous for *Eps-A*^*m*^*1* flanking genes *MOT1* (*MODIFIER OF TRANSCRIPTION 1*) and *SMP* (*SM-LIKE PROTEIN*). The isogenic background of these lines reduced genetic variability and facilitated the precise mapping of the *Eps-A*^*m*^*1* locus as a Mendelian trait rather than as a quantitative trait locus (QTL). Young seedlings were screened with molecular markers for *MOT1* and *SMP*, and only plants carrying recombination events between these two markers were phenotyped.

Previous studies have shown that the differences in flowering time between the *Eps-A*^*m*^*1-e* and *Eps-A*^*m*^*1-l* alleles can be maximized by exposing the plants to fluorescent lights at a low fluency (160 μM m^−2^ s^−1^), a long-day photoperiod (16 h of light and 8 h of darkness), and constant cool temperatures (16 °C) (Lewis et al. 2008). The same environmental conditions were used in this study to phenotype the progeny tests of the plants carrying the closest recombination events to *Eps-A*^*m*^*1.* Some preliminary experiments were performed under greenhouse conditions (20–25 °C, natural day length supplemented with incandescent lamps to extend photoperiod to 16 h), where the differences were smaller but still significant. Heading times were registered from sowing to spike emergence.

Molecular markers used for the construction of the high-density genetic map are listed in Supplementary Table S1. DNA extraction and PCR procedures were performed as described before (Kippes et al. 2014). Markers were developed using BAC sequences from the *T. monococcum* physical map (see next section) and sequences from wheat genes homologous to *Brachypodium distachyon* genes located in the region colinear to *Eps-A*^*m*^*1* (Faricelli et al. 2010). *Eps-A*^*m*^*1* flanking genes *FTSH4* (*FTSH PROTEASE 4*) and *SMP* were used to search a genomic database of *B. distachyon* (http://www.phytozome.net) and establish the colinear target region. The genes detected in this region were then used as queries to search a *T. aestivum* L. (2*n* = 2*x* = 42, AABBDD) database of flow-sorted chromosomes arms developed by the International Wheat Genome Sequencing Consortium (IWGSC 2014, http://www.wheatgenome.org/). A reverse BLASTP search from the best wheat candidate to the *Brachypodium* proteome was done, to confirm that the correct homolog was found.

### Physical map

A BAC library from *T. monococcum* ssp. *monococcum* accession DV92 (Lijavetzky et al. 1999) was screened with PCR markers developed for the *Eps-A*^*m*^*1* region (Supplementary Table S2). DNA was isolated from the selected BAC clones using the QIAGEN® Large-Construct Kit (Qiagen, USA) and then fragmented in a Covaris ultrasonicator (peak power, 175; duty factor, 10 %; cycles/burst, 200; time, 110 s). Libraries for Illumina sequencing were prepared using the KAPA LTP Library Preparation Kit Illumina platforms (KAPA Biosystems, USA). The quality of the libraries was assessed in an Agilent 2100 Bioanalyzer instrument using the High Sensitivity DNA Kit (Agilent Technologies, USA). Libraries were sequenced using 100-bp paired-end reads on Illumina HiSeq2000. Individual BAC coverage was higher than 100×.

Sequence assembly was performed using CLC 6.5 Beta 4 software (CLC-Bio, USA). Once sequences were assembled, repetitive elements were identified using the Triticeae Repeat Sequence Database (http://wheat.pw.usda.gov/ITMI/Repeats/blastrepeats3). The nonrepetitive sequences were then annotated using a combination of tools, including comparative genomics analyses, BLAST searches of other annotated grass genomes, and the annotation pipeline Triannot (Leroy et al. 2011). Nonrepetitive sequences of the newly sequenced BACs were used to develop new primers to rescreen the BAC library. This process was reiterated several times to expand the physical map of the *Eps-A*^*m*^*1* region. Individual BAC sequences have been deposited in GenBank (Supplementary Table S3).

### Characterization of *Eps-A*^*m*^*1* candidate genes and its potential targets

Candidate genes completely linked to *Eps-A*^*m*^*1* in the high-density genetic map were amplified from both parental lines of the *T. monococcum* mapping population (DV92 and G3116) and sequenced. GenBank accession numbers are listed in Supplementary Table S3; primers used are listed in Supplementary Table S4. The predicted protein products of the three candidate genes were compared between the parental lines using BLASTP. Amino acid polymorphisms are described using a letter indicating the amino acid in the *T. urartu* (PI 428198) protein, followed by the position of that amino acid from the initial methionine in the *T. urartu* reference protein (Supplementary Figs. S1–S3), and by a letter describing the derived amino acid. For the three candidate proteins characterized in this study, *T. urartu* carries the ancestral state in all the analyzed amino acid positions. Therefore, in this study, the first letter in a polymorphism corresponds to the ancestral stage and the last letter to the derived stage. Ancestral stages were inferred from the alignment of the predicted wheat proteins with the corresponding orthologs from *Hordeum vulgare*, *B. distachyon*, *Sorghum bicolor*, *Setaria italica*, *Oryza sativa*, *Zea mays*, and Arabidopsis (Supplementary Figs. S1–S3). Multiple alignments were performed using ClustalW2 (http://www.ebi.ac.uk/Tools/msa/clustalw2/). The potential effect of amino acid changes was predicted using multiple tools including BLOSUM62 matrix scores (Henikoff and Henikoff 1992), PROVEAN scores (http://provean.jcvi.org; Choi et al. 2012), and PolyPhen scores (http://genetics.bwh.harvard.edu/pph2/index.shtml; Adzhubei et al. 2010).

The expression levels of the *Eps-A*^*m*^*1* candidate genes and some of the known targets of the main candidate protein were characterized using quantitative reverse transcription PCR (qRT-PCR). *T. monococcum* NILs carrying the *Eps-A*^*m*^*1-e* (early flowering) and the *Eps-A*^*m*^*1-l* (late flowering) alleles were grown in a growth chamber under continuous temperature (16 °C) and long-day photoperiod (16 h fluorescent lights at 160 μM m^−2^ s^−1^). Five weeks after sowing, when plants carrying the *Eps-A*^*m*^*1-e* allele were at the terminal spikelet stage and plants carrying the *Eps-A*^*m*^*1-l* allele were at the double ridge stage, tissue was collected from young leaves every 4 h during a 24-h period. Six individual plants were sampled at each time point.

RNA samples were extracted using the Spectrum Plant Total RNA Kit (Sigma-Aldrich). First-strand cDNAs were synthesized from 1 μg of total RNA using the High Capacity Reverse Transcription kit (Applied Biosystems). Quantitative PCR was performed using SYBR Green and a 7500 Fast Real-Time PCR system (Applied Biosystems). Primers used for SYBR GREEN quantitative PCR are listed in Supplementary Table S5. *ACTIN* was used as an endogenous control. Transcript levels for all genes are expressed as linearized fold-*ACTIN* levels calculated by the formula 2^(*ACTIN* CT − *TARGET* CT)^ ± standard error (SE) of the mean. The resulting number indicates the ratio between the initial number of molecules of the target gene and the number of molecules of *ACTIN*.

### Screening of a tetraploid wheat TILLING population

The complete coding regions of the A- and B-genome copies of candidate gene *ELF3* were obtained from available sequences from *T. aestivum* cultivar Chinese Spring (IWGSC 2014, http://www.wheatgenome.org), and from *T. turgidum* ssp. *durum* cultivar Kronos transcriptome (Krasileva et al. 2013; http://wheat.pw.usda.gov/GG2/WheatTranscriptome/). Genome-specific primers were developed (Supplementary Table S6) and tested using Chinese Spring nullisomic-tetrasomic lines N1AT1D and N1BT1D, and confirmed by DNA sequencing.

A screening of DNAs from 1536 TILLING lines was performed using 384 pools, each including four DNAs. PCR amplification products of the target region were digested with *Cel*I, as described before (Uauy et al. 2009). Individual mutant lines were identified within the selected pools, and the PCR amplification products were sequenced to characterize the mutations. Lines carrying mutations that introduced premature stop codons in the A- or the B-genome copies of the candidate gene *ELF3* were selected and were then intercrossed to combine the mutations in both homoeologs. The resulting lines were backcrossed to the non-mutagenized variety Kronos for two generations to reduce the number of background mutations. Mutations were selected by sequencing in each backcross generation. BC_2_ plants heterozygous for mutations in the two *ELF3* homoeologs were selected and self-pollinated. Plants with no functional copies of *ELF3* (*elf3*-null) and control plants homozygous for the wild-type *ELF3* alleles (*ELF3*-WT) were selected from the segregating BC_2_F_2_ plants.

### Epistatic interactions between *ELF3* and *PPD1*

The tetraploid *elf3*-null lines were also crossed to a Kronos near-isogenic line carrying the wild-type photoperiod-sensitive *PPD-A1b* allele, developed before (Pearce et al. 2013). A BC_2_ line heterozygous for *ELF3* and *PPD-A1* was identified using molecular markers and was self-pollinated to produce a BC_2_F_2_ population segregating for both genes. A screening of the segregating population identified lines homozygous for the four possible allelic combinations: *ELF3-*WT*/PPD-A1a*, *ELF3-*WT*/PPD-A1b*, *elf3*-null*/PPD-A1a*, and *elf3-*null*/PPD-A1b.* Primers used to genotype *ELF3* are listed in Supplementary Table S6. Markers used for *PPD-A1* have been described before (Wilhelm et al. 2009).

BC_2_F_3_ plants carrying the four allelic combinations were grown under both SD (8 h light) and LD (16 h light) photoperiods using fluorescent lights and a constant temperature of 16 °C. Heading time and number of spikelets per spike were determined for each plant, and the effects of the individual alleles and their epistatic interactions were statistically evaluated using two by two factorial ANOVAs. Statistical analyses were performed using SAS program version 9.4.

### Introgression of the *Eps-A*^*m*^*1-l* locus into tetraploid wheat

The chromosome region including *Eps-A*^*m*^*1* from *T. monococcum* accession DV92 (2*n* = 14, A^m^A^m^) was introgressed into *T. turgidum* ssp. *durum* cultivar CBW0112 (2*n* = 28; AABB). To generate this material, we first intercrossed *T. turgidum* ssp*. durum* cultivar Langdon and *T. monococcum* ssp. *monococcum* cultivar DV92 and treated the ABA^m^ hybrid with colchicine to double the chromosome number. We backcrossed the resulting AABBA^m^A^m^ amphyploid to CBW0112 for five generations, selecting the *Eps-A*^*m*^*1* allele in each crossing cycle with flanking markers *wg241* and *NDK3* (*NUCLEOSIDE DIPHOSPHATE KINASE 3*) (Supplementary Table S1). A schematic representation of the process is presented in Supplementary Fig. S4.

CBW0112 BC_5_F_3_ near-isogenic lines homozygous for the *Eps-A*^*m*^*1* segment from DV92 were selected for phenotypic characterization. Sister lines homozygous for the *Eps-A1* allele from the A-genome of tetraploid wheat were used as negative controls. Lines were grown under controlled conditions (16 °C constant temperature, 16 h fluorescent lights), and heading time and spikelet number were registered.

When lines were at BC_3_F_2_, seven SSR markers and three CAP markers previously mapped to wheat chromosome 1A were tested to determine the size of the introgressed 1A^m^ chromosome segment (Supplementary Table S7, Supplementary Fig. S4).

## Results

### Redefinition of the target chromosome region for *Eps-A*^*m*^*1*

**Fig. 1:**
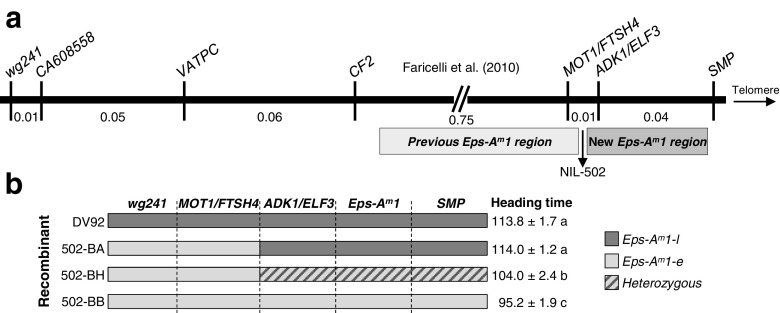
**a** *T. monococcum* high-density genetic map based on the analysis of 10,000 gametes, showing the previous candidate region for *Eps-A* ^*m*^ *1* in *light gray* (Faricelli et al. 2010). The reevaluation of critical NIL 502 resulted in a new candidate region for *Eps-A* ^*m*^ *1*, indicated in *darker gray*. **b** Graphical genotypes and heading times of a progeny test of critical NIL 502. *Different colors* indicate chromosome regions homozygous for the DV92 allele (*dark gray*), homozygous for the G3116 allele (*light gray*), or heterozygous (*diagonal gray lines*). Heading time for each line is shown as the mean of at least five plants ± standard error of the mean. *Values followed by different letters* are significantly different from each other (*P* < 0.01). This progeny test confirmed that the *Eps-A* ^*m*^ *1* locus in NIL 502 was distal to the *MOT1/FTSH4* locus

**Fig. 2:**
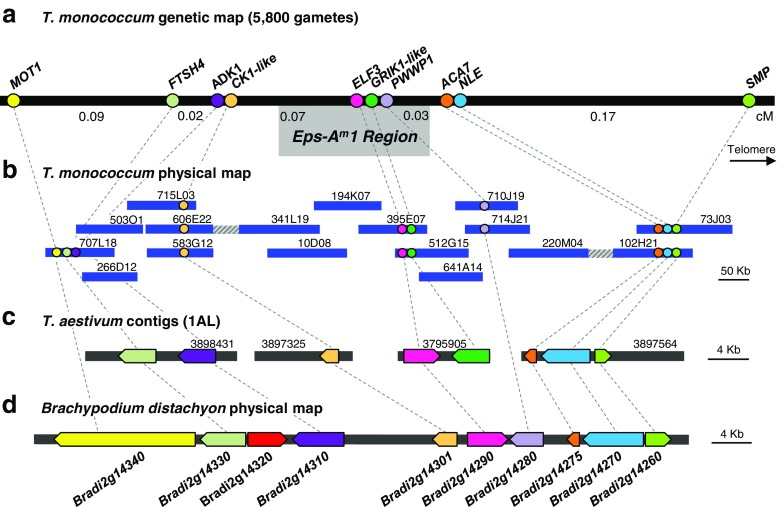
**a** *Triticum monococcum* high-density genetic map. Genetic distances are based on the analysis of 5800 gametes. Putative genes are color-coded and indicated as *circles*. **b** *Triticum monococcum* physical map. Sequenced BACs are indicated as *blue bars* and assembly gaps as *striped bars*. **c** *Triticum aestivum* 1AL IWGSC contigs colinear to the *Eps-A* ^*m*^ *1* region. Contig names were shortened, and only the contig number is shown. **d** *Brachypodium distachyon* region colinear to *Eps-A* ^*m*^ *1*

In a previous study, the *T. monococcum Eps-A*^*m*^*1* locus was mapped linked to genes *MOT1* and *FTSH4* (Faricelli et al. 2010; Fig. 1a). Two years later, two independent studies in barley identified *ELF3* as the gene responsible for the *eam8* mutation (Faure et al. 2012; Zakhrabekova et al. 2012), which maps very close to *MOT1* and *FTSH4*. Since both *Eps-A*^*m*^*1* and *eam8* loci affect similar traits (Lewis et al. 2008; Faure et al. 2012) and are physically close to each other (only 28 kb apart in *Brachypodium*), we decided to add *ELF3* to our previous map and revisit the critical recombination events (Fig. 2a, b).

We sequenced *ELF3* in the parental lines DV92 (AC270217.1) and G3116 (GenBank KU570055) and used one of the discovered SNPs to develop a codominant marker (Supplementary Table S1). Using this marker, we mapped *ELF3* completely linked to *ADK1* (similar to rice putative kinase *ADK1*, AAT44307) in the same *T. monococcum* segregating population used before to map *Eps-A*^*m*^*1* (Fig. 1a, Faricelli et al. 2010). In this population, a single recombination event in NIL 502 (Fig. 1a, b) separated *MOT1/FTSH4* from *ADK1/ELF3*. NIL 502 carries the G3116 alleles for *MOT1/FTSH4* and the DV92 alleles for *ADK1/ELF3* (Fig. 1b), so if this line flowers early it would indicate that *Eps-A*^*m*^*1* is linked to *MOT1/FTSH4* and if it flowers late it would indicate that *Eps-A*^*m*^*1* is linked to *ADK1/ELF3.*

NIL 502 was initially classified as an early flowering line (Lewis et al. 2008), but a reexamination of the original progeny test showed that it had an intermediate flowering time when compared to the early and late heading NILs. To determine if the intermediate flowering time was the result of more than one gene affecting flowering segregating in this region or simply the pleiotropic effect of other genes still segregating in the genetic background of this line, we backcrossed NIL 502 twice to a NIL carrying the G3116 alleles for all markers in this region. The resulting BC_2_F_2_ plants were homozygous G3116 for *MOT1/FTSH4* and segregated for the distal *ADK1/ELF3* genes (Fig. 1b). These lines showed a clear segregation for heading time that was perfectly associated to the *ADK1/ELF3* alleles (Fig. 1b). In addition, the homozygous recombinant lines showed almost identical flowering time as the parental line DV92 (Fig. 1b). These two results demonstrated that NIL 502 carries the *Eps-A*^*m*^*1* allele for late flowering and that, therefore, *Eps-A*^*m*^*1* is linked to *ADK1/ELF3* rather than to *MOT1/FTSH4* as suggested before (Faricelli et al. 2010). *Erratum* notes are being submitted to the original papers correcting the phenotyping of NIL 502 (Lewis et al. 2008; Faricelli et al. 2010).

### High-density genetic map of *Eps-A*^*m*^*1* in *T. monococcum*

To further characterize the relationship between *Eps-A*^*m*^*1* and *ELF3*, we developed a new high-density genetic map for the region between *MOT1* and *SMP* (Fig. 2a). Using these two flanking markers, we screened 2900 BC_6_F_2_ NILs (5800 gametes) and identified 22 lines with recombination events in the target region. Based on the heading time of BC_6_F_3_ progenies of the selected recombinants grown under controlled environment conditions, the *Eps-A*^*m*^*1* locus was mapped 0.20 cM proximal to *SMP* and 0.18 cM distal to *MOT1* (Fig. 2a). The 10 recombination events found between *MOT1/FTSH4* and *Eps-A*^*m*^*1* clearly demonstrated that neither *MOT1* nor *FTSH4* are viable candidate genes for *Eps-A*^*m*^*1*. This result is consistent with the conclusion obtained from the reanalysis of the progeny of the critical NIL 502 reported above.

The *ELF3* gene was completely linked to the flowering phenotype in all the recombinant lines, suggesting that it is a viable candidate gene for *Eps-A*^*m*^*1.* To further delimit the *Eps-A*^*m*^*1* candidate region, we designed additional markers based both on *B. distachyon* genes flanking *ELF3* and on nonrepetitive sequences obtained from the *T. monococcum* BACs identified during the construction of the physical map (see next section, Fig. 2b).

The *B. distachyon* orthologs of wheat *Eps-A*^*m*^*1* flanking genes *FTSH4* (*Bradi2g14330*) and *SMP* (*Bradi2g14260*) define a 47-kb genomic region including the *Brachypodium* orthologs of *ADK1* (*Bradi2g14310*), *ELF3* (*Bradi2g14290*), and five additional annotated genes (Table 1 and Fig. 2d). These *Brachypodium* genes were used as queries to identify the closest homologs in the *T. aestivum* draft genome sequence (IWGSC 2014). Three wheat orthologs were identified using this strategy, in addition to *ADK1* and *ELF3*, and were mapped to the target region (Fig. 2c). The wheat ortholog of *Bradi2g14280* was not detected in the *T. aestivum* database but was later found in one of the BAC sequences and was incorporated into the high-density genetic map (Fig. 2). The closest wheat homolog of *Bradi2g14320* was found in wheat chromosome 2AS and, therefore, was eliminated from further analyses. One additional gene, designated as *GRIK1-like*, was found in the same *T. aestivum* contig carrying the *ELF3* gene (IWGSC-contig 3795905), but was not present in the *Brachypodium* colinear region*. GRIK1-like* encodes a serine/threonine-protein kinase similar to GRIK1 (GEMINIVIRUS REP INTERACTING KINASE 1) from Arabidopsis.

**Table 1:** Summary of genes found in *Brachypodium distachyon* and *T. aestivum*, in the colinear *Eps-A* ^*m*^ *1* region defined between flanking genes *FTSH4* (*Bradi2g14330*) and *SMP* (*Bradi2g14260*). Wheat chromosome location (A-genome) and contig identification numbers correspond to the best hit detected using the blast tool at www.wheatgenome.org. Contig names were shortened to retain the contig number only. *Brachypodium* and *T. aestivum* gene names were obtained from http://plants.ensembl.org

Gene	*Brachypodium* gene	*T. aestivum* chr.	*T. aestivum* IWGSC contig	*T. aestivum* gene	*Brachypodium* gene description
*GRF11*	*Bradi2g14320*	2AS	5298309	Traes_2AS_CA4D79100	Growth-regulating factor 11-like
*ADK1*	*Bradi2g14310*	1AL	3898431	Traes_1AL_FC50A1181	Casein kinase I-like
*CK1-like*	*Bradi2g14301*	1AL	3897325	Traes_1AL_744933633	Casein kinase I isoform delta-like
*ELF3*	*Bradi2g14290*	1AL	3795905	Traes_1AL_52C5531A4	EARLY FLOWERING 3-like
*GRIK1-like*	*Bradi1g11340* ^a^	1AL	3795905	Traes_1AL_A78FD4ED4	Serine/threonine-protein kinase GRIK1-like
*PWWP1*	*Bradi2g14280*	1AL	–	–	PWWP domain-containing protein
*ACA7*	*Bradi2g14275*	1AL	3897564	–	Alpha carbonic anhydrase 7-like
*NLE*	*Bradi2g14270*	1AL	3897564	Traes_1AL_D14E918FD	Notchless protein homolog

^a^Not present in the *Brachypodium* colinear region

After the inclusion of the additional markers in the high-density genetic map, the *Eps-A*^*m*^*1* region was delimited to a 0.1-cM interval flanked by *ADK1/CK1-like* (*CASEIN KINASE I ISOFORM DELTA-LIKE*) on the proximal side and *ACA7* (*ALPHA CARBONIC ANHYDRASE 7-LIKE*) on the distal side. In the high-density mapping population, *Eps-A*^*m*^*1* was completely linked to *ELF3*, *GRIK1-like*, and *PWWP1* (PWWP domain-containing protein) (Fig. 2a), which are described briefly below and in more detail in the “Discussion” section.

*ELF3* is a circadian clock gene involved in the regulation of flowering time in Arabidopsis, rice, legumes, and barley (Hicks et al. 2001; Matsubara et al. 2012; Saito et al. 2012; Weller et al. 2012; Faure et al. 2012; Zakhrabekova et al. 2012). *GRIK1-like* encodes a putative serine/threonine kinase that is homologous to Arabidopsis *GRIK1.* This Arabidopsis gene is part of a cascade that coordinates the metabolic requirements of rapidly growing cells and geminivirus-infected cells (Shen et al. 2009). *PWWP1* encodes a putative PWWP domain-containing protein with no clear homologs in Arabidopsis. This domain is found in proteins that are involved in cell division, growth, and differentiation, but the actual function of this gene is currently unknown. Among the three *Eps-A*^*m*^*1* candidate genes, we prioritized *ELF3* for further characterization based on its known role in the regulation of flowering in several plant species.

### Physical map of the *Eps-A*^*m*^*1* region

A BAC library from *T. monococcum* accession DV92 including 276,000 clones (5.6-fold coverage, Lijavetzky et al. 1999) was screened with markers for flanking genes *FTSH4* and *SMP* and for the linked candidate gene *ELF3*.

The chromosome walk from the proximal site started from the distal border of BAC 707L18, which included genes *MOT1*, *FTSH4*, and *ADK1.* Six BACs were mapped to this region, covering ~270 kb. Gene *CK1-like*, an ortholog of *Bradi2g14301*, was also detected in this region (Fig. 2b).

On the distal side of *Eps-A*^*m*^*1*, a screening using primers for the *SMP* gene yielded BACs 102H21 and 73J03, which covered ~197 kb. Genes *SMP*, *ACA7*, and *NLE* (*NOTCHLESS*), orthologous to *Bradi2g14260*, *Bradi2g14270*, and *Bradi2g14275*, were detected in close proximity, within an 8.6-kb region present in both BACs (Fig. 2b). Although the physical distance between genes *NLE* and *SMP* is only of 346 bp, we detected 10 recombination events between them. The analysis of the sequence between these two genes revealed the presence of trinucleotide repeats, which have been associated before to fragile sites and chromosomal instability hot spots that can lead to high recombination frequencies (Aguilera and Gómez-González 2008).

A third screening of the *T. monococcum* BAC library was performed using PCR markers for *ELF3*, which was completely linked to *Eps-A*^*m*^*1*. Two BACs were sequenced, and from their borders, chromosome walks were initiated in both directions. A total of nine BACs were mapped to this contig, covering ~570 kb. Genes *ELF3*, *GRIK1-like*, and *PWWP1* were identified in this region (Fig. 2b).

Nine of the 10 genes annotated in the colinear region in *Brachypodium* are included in the *T. monococcum* physical map, and the last one (*Bradi2g14320*) has its closest homolog in a different wheat chromosome. Since all the genes annotated in the colinear region in *Brachypodium* have been accounted for, any additional wheat genes found in the two gaps still present in the *T. monococcum* physical map (Fig. 2b) are not expected to be colinear with *Brachypodium*.

In summary, we constructed and sequenced an ~1-Mb physical map of the *MOT1*-*SMP* region, which includes 10 wheat genes. The resulting gene density of roughly one gene per 100 kb found in the *Eps-A*^*m*^*1* region is very similar to the average of one gene per 96 kb found before for an 18.2-Mb region in wheat chromosome 3B (Choulet et al. 2010). The *T. monococcum* genes were distributed in small islands including one to three genes separated by large regions of repetitive elements, which is similar to distributions found in other wheat genome regions (Gottlieb et al. 2013).

### Characterization of *Eps-A*^*m*^*1* candidate proteins

To help us prioritize or discard any of the three *T. monococcum* candidate genes completely linked to *Eps-A*^*m*^*1*, we compared the predicted protein sequences encoded by the parental lines, DV92 and G3116, using primers listed in Supplementary Table S4. We also analyzed their expression profiles in NILs carrying the DV92 or G3116 *Eps-A*^*m*^*1* alleles using the qRT-PCR primers listed in Supplementary Table S5.

The predicted ELF3 protein showed four polymorphisms between the two parental lines: V364L, G681R, G700D, and G718A, which are predicted to have intermediate to large effects on protein function based on either BLOSUM62, PROVEAN, or PolyPhen-2 scores (Table 2). None of these polymorphisms were located within the conserved blocks defined by Liu et al. (2001) (Supplementary Fig. S1). Comparison of the *T. monococcum* sequences with homologous proteins from other species indicates that DV92 has the derived amino acids for the V364L and G681R polymorphisms and G3116 has the derived amino acids at G700D and G718A (Supplementary Fig. S1). The ancestral ELF3 haplotype (haplotype-A) is present in *T. urartu*, in the A- and B-genome of tetraploid wheat and, with single amino acid changes, in the D-genome of hexaploid wheat, barley, *Brachypodium*, and sorghum (Supplementary Fig. S1 and Table S8). The G3116 haplotype (haplotype-B) was frequent among *T. monococcum* ssp. *aegilopoides* accessions and among *T. monococcum* ssp. *monococcum* accessions with a winter growth habit (Supplementary Table S8), whereas the DV92 haplotype (haplotype-C) was predominant among *T. monococcum* ssp. *monococcum* accessions with a spring growth habit. Interestingly, five out of the seven *T. monococcum* ssp. *monococcum* accessions carrying ELF3 haplotypes A or B were collected in Turkey, where this species was initially domesticated (Heun et al. 1997).

**Table 2:** Amino acid polymorphisms in candidate genes *ELF3*, *GRIK1-like*, and *PWWP1* between cultivated *T. monococcum* ssp. *monococcum* accession DV92 carrying the *Eps-A* ^*m*^ *1-l* allele for late heading, and wild *T. monococcum* ssp. *aegilopoides* accession G3116 carrying the *Eps-A* ^*m*^ *1-e* allele for early heading. Amino acid changes are described indicating the ancestral amino acid first, followed by the position of that amino acid from the initial methionine in the *T. urartu* (PI 428198) protein, and the derived amino acid at the end. Ancestral and derived states are inferred from other grasses listed in Supplementary Table S8. PROVEAN, PolyPhen, and BLOSUM62 scores were estimated for the change from the ancestral to the derived variant. Scores predicting a disruptive effect on protein structure and function are indicated in italics

Gene	Amino acid change	DV92 allele	G3116 allele	PROVEAN^a^	PolyPhen-2^b^	BLOSUM62^c^
*ELF3*	V364L	L	V	−0.688	0.574	1
	G681R	R	G	*−2.781*	0.116	*−2*
	G700D	G	D	1.046	*0.999*	*−1*
	G718A	G	A	0.613	0.114	0
*GRIK1-like*	D77E	D	E	0.389	0.012	2
	A228S	A	S	0.298	0.048	1
	D351N	D	N	−0.039	0.016	1
*PWWP1*	S251W	S	W	−2.023	*0.992*	*−3*
	T294M	M	T	0.653	0.050	*−1*
	S327I	S	I	−1.703	*0.827*	*−2*
	G542C	G	C	−0.540	*0.999*	*−3*
	V701A	A	V	0.387	0.455	0

^a^PROVEAN scores were calculated at provean.jcvi.org. Scores <−2.5 are predicted to have a strong effect on protein function

^b^PolylPhen-2 scores were calculated using genetics.bwh.harvard.edu/pph2/. Values close to 0 suggest limited effects on protein function, and values closer to 1 suggest more significant effects on protein structure and function

^c^BLOSUM62 scores were obtained from BLOSUM62 substitution matrix (Henikoff and Henikoff 1992). The more negative the BLOSUM62 scores are, the higher is the probability of that amino acid change to disrupt protein structure or function

To analyze the linkage between ELF3 haplotypes and *Eps1* alleles, we evaluated a segregating BC_1_F_2_ population generated by crossing *T. monococcum* ssp. *monococcum* accession PI 355522 (ELF3 haplotype-B) to DV92 (ELF3 haplotype-C) and then backcrossing the F1 to DV92. These lines were grown in a controlled environment (16 °C and 16 h of light), and heading time and spikelet number were registered. This experiment confirmed that PI 355522 carries the *Eps-A*^*m*^*1-e* allele for early flowering and reduced number of spikelets (Supplementary Table S9).

In a separate experiment, we characterized 10 additional *T. monococcum* accessions from Supplementary Table S8 under saturated vernalization (6 weeks at 4 °C) and photoperiod conditions (16 h fluorescent light). Under these conditions, we found that plants from the four *T. monococcum* accessions carrying the ELF3 haplotype-B headed more than 1 month earlier than plants from the six accessions carrying the ELF3 haplotype-C (Supplementary Table S10). These results suggest that the four haplotype-B lines carry the *Eps-A*^*m*^*1-e* allele and the six haplotype-C lines carry the *Eps-A*^*m*^*1-l*, but this linkage was not tested in this study.

The GRIK1-like protein showed three polymorphisms between G3116 and DV92 (D77E, A228S, and D351N, Supplementary Table S8), which are all predicted to have limited effects on protein function based on BLOSUM62, PROVEAN, and PolyPhen-2 scores (Table 2). Polymorphisms A228S and D351N are both located within the serine/threonine protein kinase catalytic domain, whereas D77E is located outside this conserved domain (Fig. S2). Comparisons with homologous proteins from other grass species indicate that DV92 carries the ancestral allele at these three amino acid positions (Supplementary Fig. S2 and Table S8). The D351N polymorphism was detected only in G3116 and *T. monococcum* ssp. *monococcum* PI 355522. The D77E and A228S polymorphisms were present in the same accessions that carry the ELF3 haplotype-B (Supplementary Table S8).

The predicted PWWP1 proteins showed two indel polymorphisms and five amino acid changes between G3116 and DV92. These changes were all located outside the conserved PWWP domain (Supplementary Fig. S3). The indels were detected in variable regions of the protein, so it was not possible to determine the ancestral and derived states. Among the five amino acid changes observed in PWWP1, DV92 has the derived state for T294M and V701A, and G3116 has the derived state for S251W, S327I, and G542C (Supplementary Fig. S3). The derived amino acid polymorphisms in G3116 are predicted by BLOSUM62, PROVEAN, and PolyPhen-2 to have larger impact on protein function than those in DV92 (Table 2). The derived amino acids in PWWP1 found in G3116 were also present in other *T. monococcum* accessions carrying the ELF3 A- and B-haplotypes but not in those carrying the ELF3 haplotype-C. All the accession from this last group showed the V701A polymorphism (Supplementary Table S8).

### Characterization of the expression profiles of *Eps-A*^*m*^*1* candidate genes

**Fig. 3:**
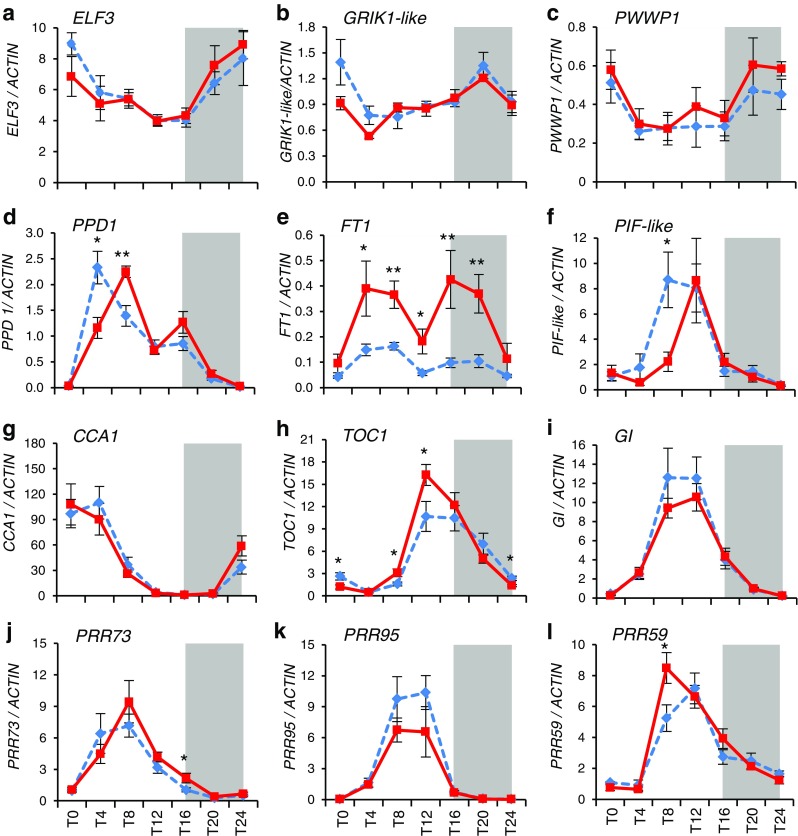
Expression analysis of candidate genes (**a–c**), flowering genes (**d–f**), and circadian clock genes (**g–l**). *T. monococcum* NILs carrying the *Eps-A* ^*m*^ *1* allele from DV92 (*Eps-A* ^*m*^ *1-l*, *blue dashed lines*) or G3116 (*Eps-A* ^*m*^ *1-e*, *red lines*) were grown under controlled conditions (16 °C; 16 h light). Each point represents means ± standard error of the mean of six individual plants (*P < 0.05, **P < 0.01, ***P < 0.001)

To test if the *Eps-A*^*m*^*1* phenotype was caused by differential expression of any of the candidate genes, transcript levels of *ELF3*, *GRIK1-like*, and *PWWP1* were measured in *T. monococcum Eps-A*^*m*^*1-e* and *Eps-A*^*m*^*1-l* NILs grown under controlled environmental conditions (Fig. 3a–c). Samples were collected from leaves because preliminary studies showed significant differences in *PPD1* and *FT1* transcript levels between *Eps-A*^*m*^*1* alleles in this tissue. Although the leaf samples were collected from plants at the same chronological age (5 weeks after sowing), the shoot apical meristems were more advanced in the early flowering NILs (*Eps-A*^*m*^*1-e* allele, terminal spikelet stage) than in the late flowering NILs (*Eps-A*^*m*^*1-l* allele, double ridge stage). No significant differences in the transcription profiles of the three candidate genes were detected between *Eps-A*^*m*^*1* alleles during a 24-h time course (Fig. 3a–c).

Among these three genes, *ELF3* showed transcript levels (Fig. 3a) that were almost an order of magnitude higher than those observed for *GRIK1-like* and *PWWP1* (Fig. 3b, c). We also analyzed the expression profiles of the A-genome homoeologs of these three genes in hexaploid wheat using previously published RNA-seq data (IWGSC 2014) and WheatExp (Pearce et al. 2015). *GRIK1-like* showed relatively uniform transcript levels across different tissues and developmental stages (Supplementary FigS5a). *PWWP1* showed low transcript levels across multiple tissues and developmental stages with a 4–8-fold increase in the last stages of grain development (Supplementary Fig. S5b). Finally, *ELF3* showed similar expression levels across tissues and developmental stages, with a 2-fold increase in the latest stage of grain development (Z85, Supplementary Fig. S5c).

### Validation of *ELF3* as a candidate gene for *Eps-A*^*m*^*1*

Studies in barley have shown that loss-of-function mutations in *ELF3* accelerate the transition from vegetative to reproductive stages and the duration of spike development in this species (Faure et al. 2012). Since these two traits were also affected by the *Eps-A*^*m*^*1* alleles, we prioritized the validation of *ELF3* as a candidate gene for *Eps-A*^*m*^*1*. These validation studies included (*i*) the comparison of the expression of known downstream targets of ELF3, (*ii*) the determination of the effect of the introgression of the *Eps-A*^*m*^*1* allele in tetraploid wheat, and (*iii*) the determination of the effect of the elimination of all functional copies of *ELF3* in tetraploid wheat.

**Fig. 4:**
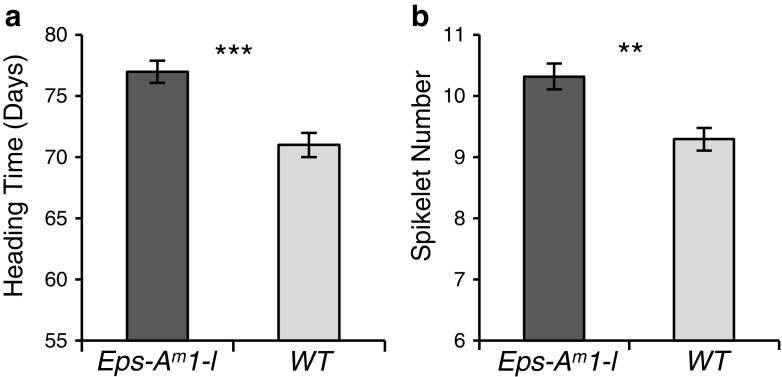
Effect of the introgression of the *T. monococcum* chromosome segment carrying the *Eps-A* ^*m*^ *1-l* allele from DV92 into tetraploid wheat on heading time (**a**) and spikelet number (**b**). *Bars* represent the mean of at least 15 plants ± standard error of the mean. *Asterisks* indicate significant differences (**P < 0.01, ***P < 0.001)
(iii) *Loss-of-function mutants of ELF3 in tetraploid wheat*: We hypothesized that if *ELF3* was the correct candidate gene for *Eps-A*^*m*^*1*, and the *ELF3* allele from DV92 was actively delaying flowering and increasing the number of spikelets per spike, then an *elf3*-null mutant should show the opposite phenotypic effects. We selected mutations that generated premature stop codons in the A and B homoeologs of *ELF3* in the TILLING population of the tetraploid wheat variety Kronos (Uauy et al. 2009). These mutations eliminate the last 241 (A-genome) or 244 (B-genome) amino acids of the C-terminal region of the ELF3 protein and are most likely loss-of-function alleles (Fig. 5a). These lines were intercrossed and then backcrossed to the recurrent parent Kronos for two generations, to reduce the number of background mutations. *ELF3*-WT and *elf3*-null homozygous BC_2_F_2_ plants (both homozygous for *PPD-A1a*) were evaluated under controlled environmental conditions (16 °C, 16 h fluorescent light). Under these conditions, the *elf3*-null Kronos plants flowered on average 7.2 days earlier than sister lines carrying wild-type *ELF3* (*P* < 0.0001; Fig. 5b) and had on average 3.8 less spikelets per spike (*P* < 0.0001; Fig. 5c). This experiment confirmed that, in tetraploid wheat, the *elf3*-null mutants have the opposite effect to the introgression of the DV92 *Eps-A*^*m*^*1-l* allele.

(i) *Expression profiles of downstream genes*: Previous studies of the barley *elf3* mutants showed significant differences in the transcription profiles of *PPD1*, *FT1*, and central circadian clock genes, so the same genes were compared between the *Eps-A*^*m*^*1* alleles. The peak of the *PPD1* expression in the NIL carrying the *Eps-A*^*m*^*1-l* allele occurred 4 h earlier (Zeitgeber time ZT4) than in the NIL carrying the *Eps-A*^*m*^*1-e* allele (ZT8), and those differences were highly significant (*P* < 0.01, Fig. 3d). The NILs carrying the *Eps-A*^*m*^*1-e* allele also showed a higher peak than *Eps-A*^*m*^*1-l* before dark, but those differences were not significant. The changes in *PPD1* were also reflected in changes in the expression of *FT1*, a downstream target of *PPD1*. NILs carrying the *Eps-A*^*m*^*1-e* showed significantly higher transcript levels of *FT1* than NILs carrying the *Eps-A*^*m*^*1-l* allele for most of the day (Fig. 3e). This result is consistent with the early flowering of the *Eps-A*^*m*^*1-e* NIL.
  Since ELF3 is involved in circadian clock regulation in both Arabidopsis and barley (Kolmos et al. 2011; Faure et al. 2012), we analyzed the expression patterns of several clock genes. Significant differences were detected in at least one time point in the *PRR* genes *TOC1* (*TIMING OF CAB EXPRESSION1*) (Fig. 3h), *PRR73* (Fig. 3j), and *PRR59* (Fig. 3l). No significant differences between *Eps-A*^*m*^*1* alleles were detected for *CCA1* (*CIRCADIAN CLOCK ASSOCIATED 1*) (Fig. 3g), *GI* (Fig. 3i), and *PRR95* (Fig. 3k). In Arabidopsis, the evening complex, formed by the ELF3, ELF4, and LUX proteins, binds to the promoters of *PIF4* and *PIF5* to repress their transcription at dusk (Nusinow et al. 2011). We identified a *T. monococcum PIF-*like gene (TmoDV92v1_076750, Fox et al. 2014) that showed a significant 4-h shift between NILs carrying the different *Eps-A*^*m*^*1* alleles (Fig. 3f). As in *PPD1*, the peak of expression of *PIF-*like occurred 4 h earlier in the NILs carrying the *Eps-A*^*m*^*1-l* allele (ZT8) than in those carrying the *Eps-A*^*m*^*1-e* allele (ZT12) (Fig. 3f).
  In summary, the set of flowering genes differentially expressed between the *Eps-A*^*m*^*1* alleles is consistent with an effect of ELF3. It should be noted that many of the changes observed in the *T. monococcum* NILs are not as severe as those reported for the *elf3* null mutants in barley. This is not an unexpected result since the barley mutations are loss-of-function mutations (Faure et al. 2012; Zakhrabekova et al. 2012), whereas the *T. monococcum ELF3* alleles differ only in four amino acid changes and are both likely functional alleles.
(ii) *Determination of the effect of the introgression of the Eps-A*^*m*^*1 allele into tetraploid wheat*: The GRIK1-like proteins encoded by the diploid *T. monococcum* DV92 and the tetraploid Kronos A-genome copy alleles are identical, but the ELF3 and PWWP1 proteins are not. Therefore, a significant effect of the introgression of the DV92 *Eps-A*^*m*^*1-l* allele into tetraploid wheat would suggest that *GRIK1-like* is not likely a good candidate gene for *Eps-A*^*m*^*1*.
  Under controlled environmental conditions (16 °C, 16 h fluorescent light), BC_5_F_3_ lines of tetraploid wheat CBW0112 carrying a distal 1A^m^ chromosome segment (Supplementary Fig. S4) including the DV92 *Eps-A*^*m*^*1-l* allele flowered 6.4 days later (*P* < 0.0001; Fig. 4a) and had 1.1 more spikelets (*P* < 0.0001; Fig. 4b) than sister lines without the *T. monococcum* introgression. These results suggest that *GRIK1-like* is an unlikely candidate gene for *Eps-A*^*m*^*1*. In addition, they suggest that the DV92 *Eps-A*^*m*^*1-l* allele includes a gene(s) that actively delays flowering time and increases the number of spikelets per spike that is effective both in diploid and tetraploid wheat.

**Fig. 5:**
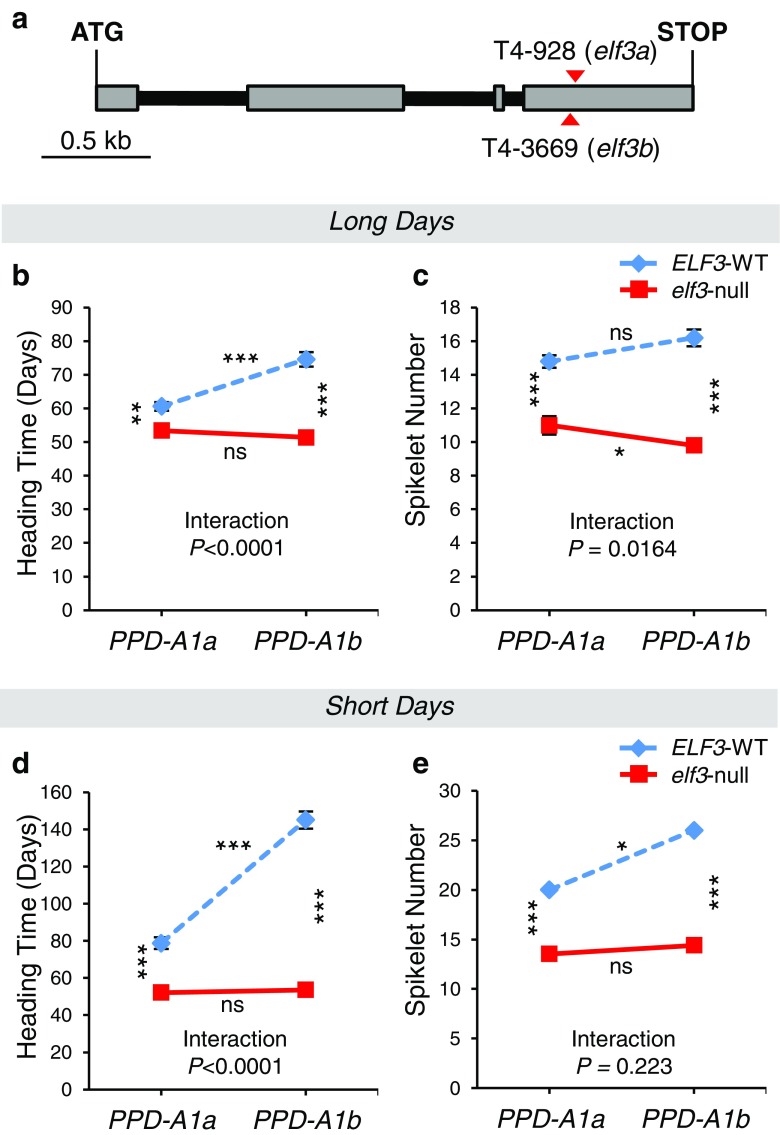
**a** Schematic representation of the *ELF3* gene. Positions of the selected mutations introducing premature stop codons on the A- and B-genome copies of the gene are indicated with *red triangles*. Exons are shown as *gray rectangles* and introns as *black lines*. **b–e** Heading time and spikelet number of tetraploid BC_2_F_3_ lines carrying four possible homozygous combinations of *ELF3* and *PPD1* alleles. Plants were grown either under LD (**b**, **c**) or SD conditions (**d**, **e**). *ELF3* alleles: *ELF3*-WT (*blue dashed lines*), *elf3*-null (*red lines*). *PPD1* alleles: *PPD-A1a* (allele with reduced photoperiodic response); *PPD-A1b* (photoperiod sensitive allele). *Asterisks between lines* indicate significant differences between *ELF3* alleles; *asterisks on top and below lines* indicate significant differences between *PPD1* alleles (*P < 0.05, **P < 0.01, ***P < 0.001, *ns* nonsignificant)

### Epistatic interactions between *ELF3* and *PPD1*

In barley, loss-of-function mutations and wild-type alleles of *ELF3* and *PPD1* showed significant epistatic interactions (Faure et al. 2012). To test if similar epistatic interactions were present in tetraploid wheat, we intercrossed the *elf3-*null line carrying the *PPD-A1a* allele for reduced photoperiodic response with a Kronos near-isogenic line carrying the photoperiod-sensitive *PPD-A1b* allele. The four different combinations of *ELF3* and *PPD-A1* homozygous alleles were selected from segregating BC_2_F_3_ plants and were evaluated under both LD and SD conditions (16 °C, fluorescent light).

Under LD, highly significant differences (*P* < 0.0001) were found between *elf3-*null plants and *ELF3-*WT sister lines both in heading time and in the number of spikelets per spike (Fig. 5b, c). For both traits, the differences between *ELF3*-alleles were larger in the presence of the photoperiod-sensitive *PPD-A1b* allele than in the presence of the *PPD-A1a* allele with reduced photoperiodic response (23.2 vs. 7.2 days and 6.4 vs. 3.8 spikelets per spike, Fig. 5b, c). These differences resulted in significant interactions between *ELF3* and *PPD1* for both heading time (*P* < 0.0001) and number of spikelets per spike (*P* < 0.05). These interactions can be visualized as nonparallel lines in the graphs presented in Fig. 5b, c. The 14-day difference in heading time between the *PPD-A1a* and *PPD-A1b* alleles (*P* < 0.0001) in the presence of the wild-type *ELF3* allele disappeared in the *elf-*null mutant background (Fig. 5b).

Under SD conditions, there was also a significant epistatic interaction for heading time between *ELF3* and *PPD1* alleles (*P* < 0.0001) but not for spikelet number (*P* = 0.2230, Fig. 5d, e). In general, the effects of the *PPD1* and *ELF3* alleles were stronger under SD than under LD. In the presence of the wild-type allele of *ELF3*, plants carrying the photoperiod-sensitive *PPD-A1b* allele flowered on average more than 66 days later than the *PPD-A1a* sister lines (Fig. 5d). When the experiment was terminated 150 days after sowing, there were still three plants with this genotype that did not flower and that were excluded from the statistical analysis. By contrast, in the presence of the *elf3*-null mutant alleles, no significant differences were observed between plants carrying the *PPD-A1a* (52.2 days) and *PPD-A1b* (53.6 days) alleles (Fig. 5d). Heading times for *elf3-*null lines under SD were comparable to those observed under LD conditions (51.4 to 53.6 days).

In summary, loss-of-function mutations in *elf3* were sufficient to abolish the differences in flowering time associated with the presence of the *PPD-A1a* or *PPD-A1b* alleles, or with different photoperiods.

## Discussion

### Characterization of the *Eps-A*^*m*^*1* candidate region

*T. monococcum* is a different species from *T. urartu*, the A-genome donor to the polyploid wheat species (Johnson and Dhaliwal 1976). These two diploid species diverged from each other before the origin of the polyploid wheat species (Dubcovsky and Dvorak 2007), so domestication and selection processes operated independently on their respective genomes. Therefore, it is not surprising that the cloning of several important agronomic genes from *T. monococcum* has revealed alleles not detected before in the polyploid wheat species. These include novel alleles for spring growth habit in the vernalization genes *VRN1* and *VRN2* (Yan et al. 2003, 2004); novel stem rust resistance genes, such as *Sr35* (Saintenac et al. 2013); and new earliness *per se* alleles, such as those detected in *Eps-A*^*m*^*3* (Gawronski et al. 2014), and in *Eps-A*^*m*^*1* in this study.

Map-based cloning approaches are easier to perform in *T. monococcum* than in the polyploid wheat species because of its diploid nature and the relatively higher levels of polymorphism (Dubcovsky et al. 1996). In this study, we used a highly polymorphic segregating population from a cross between a cultivated (DV92) and a wild *T. monococcum* accession (G3116) to map *Eps-A*^*m*^*1* within a 0.1-cM interval in the distal region of *T. monococcum* chromosome arm 1A^m^L (Fig. 2). A large deletion in the colinear region of chromosome arm 1DL was also associated with differences in earliness *per se* in hexaploid wheat (Zikhali et al. 2015). In barley, 85 out of the 195 early flowering mutants characterized by the Scandinavian barley mutation program (Lundqvist 2014) belong to the *mat-a* group (=*eam8*), that was mapped on the distal region of chromosome arm 1HL colinear with *Eps-A*^*m*^*1* (Zakhrabekova et al. 2012; Faure et al. 2012). Taken together, these results indicate the presence of a conserved earliness *per se* gene in the distal region of the long arm of homoeologous group one in both barley and wheat.

The sequencing of the *Eps-A*^*m*^*1* candidate region revealed 10 *T. monococcum* genes, including nine that were present in the colinear region in *B. distachyon* (Fig. 2). The only other *B. distachyon* gene present in this region was homologous to a wheat gene located in a different chromosome region, suggesting that the current list of *T. monococcum* candidate genes includes all the conserved genes present in this region. However, we cannot rule out the possibility of noncolinear wheat genes in the two unsequenced gaps of the current *T. monococcum* physical map. Among the 10 *T. monococcum* genes detected in this region, three were completely linked to *Eps-A*^*m*^*1*: *GRIK1-like*, *PWWP1*, and *ELF3* (Fig. 2). These three candidate genes are described in more detail below.

### *Eps-A*^*m*^*1* candidate genes

None of the three linked genes showed significant transcriptional differences between the *T. monococcum* NILs carrying the *Eps-A*^*m*^*1-l* and *Eps-A*^*m*^*1-e* alleles (Fig. 3a–c), suggesting that the differences in heading time and spikelet number between these alleles are more likely associated to the observed differences in their respective proteins. Therefore, we present below a detailed discussion of the different amino acid polymorphisms identified in this study.

#### *GRIK1-like*

The putative serine/threonine kinase encoded by this gene has been proposed to be part of a gene network that coordinates the metabolic requirements of rapidly growing cells (Shen et al. 2009). For all three amino acid polymorphism identified in GRIK1-like, the cultivated *T. monococcum* accession DV92 carries the ancestral haplotype. Since the predicted GRIK1-like protein in DV92 is identical to the one predicted for the A-genome of tetraploid wheat (Supplementary Fig. S2 and Table S8c), the significant effect of the *Eps-A*^*m*^*1-l* introgression into tetraploid wheat suggests that *GRIK1-like* is an unlikely candidate gene for *Eps-A*^*m*^*1*.

#### PWWP1

Not much is known about the PWWP1 protein except for the presence of the PWWP domain (pfam 00855). This domain is found in numerous proteins involved in cell division, growth, and differentiation. It binds to Histone-4 methylated at lysine-20, H4K20me, suggesting that it is a methyl-lysine recognition motif. The PWWP1 proteins encoded by the DV92 and G3116 alleles differ in five amino acids. The S251W and T294M polymorphisms are unlikely causal mutations for *Eps-A*^*m*^*1* because they are not polymorphic in the DV92 × PI 355522 population, which shows a clear segregation for heading time and spikelet number (Supplementary Table S9). The S327I and G542C polymorphisms are also unlikely causal mutations for *Eps-A*^*m*^*1* because these two amino acids are identical between DV92 and the A-genome of tetraploid wheat, and still the introgression of the DV92 *Eps-A*^*m*^*1*allele into tetraploid wheat is associated with significant differences in heading time and number of spikelets per spike (Fig. 4). We currently have no evidence to rule out V701A as a candidate polymorphism for *Eps-A*^*m*^*1*. However, this change is predicted to have a limited effect on protein structure and function (Table 2) and, therefore, is an unlikely causal polymorphism for the observed phenotypic differences.

#### ELF3

In Arabidopsis, ELF3 mediates the circadian gating of light responses and regulates light input to the clock (McWatters et al. 2000; Covington et al. 2001). In this species, ELF3, ELF4, and LUX form a trimeric protein complex named evening complex that directly represses circadian clock genes *PRR7* and *PRR9* (Dixon et al. 2011; Helfer et al. 2011; Kolmos et al. 2011; Chow et al. 2012; Herrero et al. 2012). The evening complex regulates the expression of growth-promoting transcription factors *PIF4* and *PIF5*, gating hypocotyl growth in the early evening (Nusinow et al. 2011). ELF3 also affects the evening loop of the circadian clock by regulating GI protein turnover (Yu et al. 2008). Mutations in *ELF3* have multiple pleiotropic effects. For example, the natural A362V mutation in Arabidopsis results in a constitutive shade avoidance phenotype and shortened circadian periods (Coluccio et al. 2011) and affects temperature-induced hypocotyl elongation (Raschke et al. 2015). Loss-of-function mutations of *elf3* result in even stronger phenotypes including arrhythmic circadian outputs under continuous light and dark and early flowering under both SD and LD photoperiods (Covington et al. 2001; Thines and Harmon 2010; Zagotta et al. 1996). Arabidopsis plants overexpressing *ELF3* show delayed flowering under LD, supporting the hypothesis that this gene acts as a flowering repressor (Liu et al. 2001).

A role of *ELF3* in the regulation of flowering is also well established in barley and supported by indirect evidence in wheat. The characterization of 87 early flowering *mat-a* mutants in barley resulted in the identification of more than 20 independent *ELF3* alleles encoding for defective proteins. These results provided convincing evidence that the early flowering of the barley *eam8*/*mat-a* mutants is caused by mutations in *ELF3* (Faure et al. 2012; Zakhrabekova et al. 2012). In hexaploid wheat, the large deletion in the distal region of the 1DL chromosome arm associated with accelerated flowering (under both SD and LD) was shown to include the *ELF-D3* gene. In addition, a S674G polymorphism in *ELF-B3* was linked to a QTL for heading time in the double haploid population of Avalon × Cadenza (Zikhali et al. 2015). These results, together with the complete linkage between *Eps-A*^*m*^*1* and *ELF3* in the *T. monococcum* high-density mapping population described in this study, provide strong support to the hypothesis that *ELF3* is *Eps-A*^*m*^*1*.

The barley *elf3-*null mutants showed an accelerated transition from vegetative to reproductive growth and accelerated spike development (Faure et al. 2012). These effects are opposite to those associated with the presence of the *T. monococcum Eps-A*^*m*^*1-l* allele. The *T. monococcum* plants carrying the *Eps-A*^*m*^*1-l* allele exhibit a delayed transition from vegetative to reproductive growth and delayed spike development that results in a significant increase in the number of spikelets per spike, both in diploid (Lewis et al. 2008) and tetraploid wheat (Fig. 4). These phenotypic differences are modulated by temperature, with stronger effects observed at 16 than at 23 °C (Lewis et al. 2008). The modulation of *Eps-A*^*m*^*1* effects by temperature provides an additional link between *Eps-A*^*m*^*1* and *ELF3* because *ELF3* plays a central role in the thermal entrainment of the clock in Arabidopsis, and the *elf3* mutant shows no evidence of temperature entrainment of the circadian clock in the dark (Thines and Harmon, 2010). In addition, a single amino acid change in the ELF3 protein was linked to variation in thermoresponsive growth in Arabidopsis through the differential regulation of *PIF4* expression and its downstream targets (Raschke et al. 2015). A complementary study showed that the binding of ELF3 to the target promoters is temperature dependent, providing a mechanism by which temperature directly controls ELF3 activity (Box et al. 2015).

### Epistatic interactions between *ELF3* and *PPD1*

*PPD1* is the major photoperiod gene in wheat (Beales et al. 2007; Wilhelm et al. 2009) and barely (Turner et al. 2005). However, the natural mutations that originated the photoperiod-insensitive allele in barley are different from the mutations that originated the reduced photoperiod-sensitive alleles in wheat. In barley, the photoperiod-insensitive *ppd-H1* carries four amino acid changes, including one in a conserved amino acid of the CCT domain, and that is the most likely causal basis of the *ppd-H1* phenotype (Turner et al. 2005). The *ppd-H1* allele is unable to accelerate flowering under long days but shows no differences with *PPD-H1* under SD. By contrast, the reduced photoperiod sensitivity mutations in wheat are associated with deletions in the promoter region that result in elevated expression of the *PPD1* gene and its downstream *FT1* target and accelerated flowering under SD (Beales et al. 2007; Wilhelm et al. 2009). Given the different nature of these *PPD1* mutations, it is interesting to compare the epistatic interactions between *ELF3* and *PPD1* in both species.

Significant epistatic interactions between *ELF3* and *PPD1* were detected both under LD and SD in wheat, but only under LD in barley (Supplementary Table S11, Faure et al. 2012). The similar effects of the barley *ppd-H1* and *PPD-H1* alleles under SD likely explain the absence of significant epistatic interactions under these conditions. By contrast, in wheat, both *PPD1* alleles have significant effects under LD and SD, although the effect under LD is largely reduced. In this study, when plants carrying the wild-type *ELF3* allele were grown under fluorescent lamps at 16 °C, the differences between the *PPD-A1a* and *PPD-A1b* alleles were 14 days, compared with 66.3 days under SD (Supplementary Table S11). However, in previous studies using stronger sodium halide lights (~260 μM m^−2^ s^−1^) and higher temperatures (day 20 °C/night 17 °C), the differences between the same *PPD1* alleles in the same Kronos background were only 4 days under LD (Chen et al. 2014). These results suggest that the light and temperature conditions selected for this study enhanced the differences between the *PPD1* alleles and likely the phenotypic differences between the *ELF3* alleles.

That an important part of the *ELF3* effect on flowering is mediated by its effect on *PPD1* is evident in the *elf3* mutants in rice. In rice, SD accelerates flowering (short-day plant) and *PPD1* (=*PRR37*) acts as a repressor of *FT-*like genes (Zhao et al. 2012; Koo et al. 2013) rather than as a promoter of flowering as observed in the long-day cereals wheat and barley. Loss-of-function mutations of *elf3* in rice result in late flowering time under both SD and LD conditions (Matsubara et al. 2012; Saito et al. 2012), opposite to the effect observed in barley and wheat. This reversion can be explained in part by the opposite effect of *PPD1*/*PRR37* in these two species, although the upregulation of the LD floral repressor *GHD7* in the rice *elf3* mutant also contributes to its late flowering. Interestingly, an amino acid change in the conserved block III of the rice ELF3 protein accelerates flowering under natural day and LD conditions, without affecting circadian rhythms (Matsubara et al. 2012). Since the evening complex interacts with multiple proteins (Huang et al. 2016), it would be interesting to test the effect of the *ELF3* natural mutations discovered in rice and in *T. monococcum* accession DV92 on these protein-protein interactions.

Although the interaction between *ELF3* and *PPD1* plays an important role in the regulation of flowering, there seems to be an additional *PPD1-*independent effect of *ELF3* on flowering time. In barley, even in the presence of the photoperiod-insensitive *ppd-H1* allele, mutations in *ELF3* result in a significant acceleration of flowering under both LD (13.3 days) and SD (25.3 days) (Supplementary Table S11, Faure et al. 2012). However, we cannot rule out the possibility that the *ppd-H1* mutations is hypomorphic and has sufficient residual effect when expressed at the increased level observed in the *eam8/mat-a* mutants (Faure et al. 2012).

### Applications and practical implications of the novel *ELF3* alleles

The significant increase in spikelet number associated with the *Eps-A*^*m*^*1-l* allele (ELF3 haplotype-C) may have favored the selection of this allele during the domestication of *T. monococcum*, explaining its high frequency among the cultivated *T. monococcum* ssp. *monococcum* accessions (Supplementary Table S8). However, since *T. monococcum* is currently cultivated in very limited regions of the world (Troccoli and Codianni 2005), we decided to transfer the *Eps-A*^*m*^*1-l* allele into tetraploid wheat. The significant increase in number of spikelets per spike (1.1 more spikelets) represents an encouraging result. However, this preliminary experiment was performed under controlled environmental conditions that are different from the ones observed in natural environments. The potential impact of this introgression on tetraploid wheat yield will require replicated field experiments in multiple environments. We are currently introgressing this *T. monococcum* chromosome segment into different tetraploid and hexaploid backgrounds to perform these experiments.

Although the results from the *Eps-A*^*m*^*1-l* allele introgression into tetraploid wheat are very preliminary for practical application, this experiment provided some valuable information. In previous studies using diploid wheat segregating populations, it was not possible to determine which of the *Eps-A*^*m*^*1* alleles represented the ancestral stage. We did not know if the *Eps-A*^*m*^*1-l* allele delayed flowering time or if the *Eps-A*^*m*^*1-e* allele accelerated the transition to the reproductive stage (Lewis et al. 2008). We speculated that the *Eps-A*^*m*^*1-l* allele was the derived state based on its high frequency among the more modern cultivated accessions of *T. monococcum*. The significant delay in heading time and increased spikelet number observed in the tetraploid lines carrying the *T. monococcum* DV92 allele support this hypothesis. It also suggests that the *Eps-A*^*m*^*1-l* has a stronger effect than the replaced *Eps-A1* allele from tetraploid wheat and that *Eps-A*^*m*^*1-l* is a hyperactive allele.

However, the results from tetraploid wheat do not provide conclusive evidence that the effects were caused by the polymorphisms in *ELF3*, because a large number of *T. monococcum* genes were introgressed together with *ELF3* into the translocated 1A^m^L chromosome segment. To provide a more conclusive answer to this question, we are mutagenizing the tetraploid line with the *T. monococcum* introgression with EMS. If we are able to identify loss-of-function mutations in the introgressed *ELF3*, and these mutations abolish the flowering delay and spikelet number increase associated with the translocated 1A^m^L chromosome segment, we will be able to conclude that the observed differences were caused by *ELF3*.

In addition to the *T. monococcum* introgression line with delayed flowering, the early flowering *elf3* tetraploid mutants generated in this study may also represent a valuable germplasm for environments that require a very short life cycle and that are exposed to very long days during their growing season. In barley, the *elf3* mutations (*eam8/mat-a*) have been used commercially in Scandinavia, in regions with very short growing seasons and long photoperiods (Lundqvist 2014). The Swedish cultivar ‘Mari’ carrying the *elf3* mutation has been grown as far north as Iceland (Lundqvist 2014). Faure et al. (2012) proposed the intriguing hypothesis that the disruption of the circadian clock observed in the *elf3* mutants may be adaptative in regions that exhibit extreme variation in light period during the year (e.g., >20 h of light per day). Although the current production of wheat at high latitudes is limited, global warming may open new geographic areas to wheat cultivation. In this context, it would be interesting to test the performance of *elf3* wheat mutants in high-latitude regions of North America, Europe, and Asia.

Even if the *elf3* loss-of-function wheat mutants find a special niche in high-latitude regions, it is unlikely that they will be useful in other regions because of the negative pleiotropic effects in the number of spikelets per spike. In our controlled environment experiments, the *elf3* Kronos mutants showed significant reductions in the number of spikelets per spike both under both LD (up to 6.4 spikelets) and SD photoperiods (up to 11.6 spikelets, Supplementary Table S1). By contrast, the *Eps-A*^*m*^*1-l* allele is associated with a positive effect on spikelet number and was selected in regions where commercial polyploid wheat species are being currently grown. This may increase the probability of this new *ELF3* allele of being adaptative in current wheat production regions. Even if this novel hyperactive *ELF3* allele is useful, it still remains to be tested if the other linked *T. monococcum* genes present in the translocated chromosome segments are associated to negative effects on agronomic performance or quality.

Control of flowering time is an important feature of plant adaptation, and the transition between the vegetative and reproductive growth needs to occur in a precise seasonal window to maximize wheat grain yield potential. The new *ELF3* alleles introduced in this study expand the genetic tools available to wheat breeders to manipulate wheat flowering time and maximize its adaptation to novel and changing environments.

## Electronic supplementary material

Below is the link to the electronic supplementary material.

ESM 1 (DOCX 479 kb)
