# Supplementary material for: Genetic and physical mapping of the earliness per se locus Eps-Am1 in Triticum monococcum identifies EARLY FLOWERING 3 (ELF3) as a candidate gene
Source: Funct Integr Genomics. 2016 Apr 16;16:365–82. doi: 10.1007/s10142-016-0490-3 (PMC4947483; doi:10.1007/s10142-016-0490-3)
Supplement: Supplementary file 1 — (DOCX 479 kb) [file 10142_2016_490_MOESM1_ESM.docx]

Electronic Supplementary Material

**Genetic and physical mapping of the earliness *per se* locus *Eps-A^m^1* in *Triticum monococcum* identifies *EARLY FLOWERING 3* (*ELF3*) as a candidate gene**

M. A. Alvarez^1^, G. Tranquilli^2^, S. Lewis^2^, N. Kippes^1^ and J. Dubcovsky ^1 *^

^1^ Department of Plant Sciences, University of California, Davis, CA 95616.

^2^ Instituto de Recursos Biológicos, INTA, Villa Udaondo (1686), Hurlingham, Buenos Aires, Argentina.

*** Corresponding author**

Jorge Dubcovsky, Professor

Department of Plant Sciences

Mail Stop 1, University of California

One Shields Avenue, Davis, CA 95616-8780

Phone: (530) 752-5159, Fax: (530) 752-4361

E-mail: jdubcovsky@ucdavis.edu

**This PDF file includes:**

Supplementary Figures S1 to S5

Supplementary Tables S1 to S11

**Supplementary Figures**

**Fig. S1** ELF3 protein alignment. Conserved blocks reported by Liu et al. (2001) are indicated in red. Black shading indicates 100% conservation. Polymorphic amino acids between DV92 carrying the late flowering *Eps-A^m^1-l* allele and G3116 carrying the early flowering *Eps-A^m^1-e* allele are highlighted in yellow. Species abbreviations and sequence IDs: *Triticum monococcum* accession DV92, TmDV (AC270217.1); *Triticum monococcum* accession G3116, TmG3 (KU570055); *Triticum urartu*, Tu-A (UCW_Tu-k25_contig_4041); *Triticum turgidum* ssp. *durum* A genome, Tt-A (UCW_Tt-k41_contig_10294); *Triticum turgidum* ssp. *durum* B genome, Tt-B (UCW_Tt-k41_contig_10295); *Triticum aestivum* D genome, Ta-D (AK332315.1); *Hordeum vulgare,* Hv (BAJ96537.1); *Brachypodium distachyon,* Bd (XP_003567779.1); *Sorghum bicolor,* Sb (XP_002440379.1); *Setaria italica,* Si (XP_004960992.1); *Oryza sativa,* Os (LOC_Os06g05060.1); *Zea mays*, Zm (NP_001169653.1); *Arabidopsis thaliana,* At (AT2G25930.1)

Block I

TmDV --MRRAGGKD---GGEDKVMGPLFPRLHVNDTTLKGGGPRAPPRNKMALYEQFSVPSQRFAANAANT-----APAAAHRPAASYAAVS--------SASAGQIGGIDRPLFPSFCVPSNE 102

TmG3 --MRRAGGKD---GGEDKVMGPLFPRLHVNDTTLKGGGPRAPPRNKMALYEQFSVPSQRFAANAANT-----APAAAHRPAASYAAVS--------SASAGQIGGIDRPLFPSFCVPSNE 102

Tu-A --MRRAGGKD---GGEDKVMGPLFPRLHVNDTTLKGGGPRAPPRNKMALYEQFSVPSQRFAANAANT-----APAAAHRPAASYAAVS--------SASAGQIGGIDRPLFPSFCVPSNE 102

Tt-A --MRRAGGKD---GGEDKVMGPLFPRLHVNDTTLKGGGPRAPPRNKMALYEQFSVPSQRFAANAANT-----APAAAHRPAASYAAVS--------SASAGQIGGIDRPLFPSFCVPSNE 102

Tt-B --MRRAGGKD---GGEDKVMGPLFPRLHVNDTTLKGGGPRAPPRNKMALYEQFSVPSQRFAANAANT-----APAAAHRPAASYAAVS--------SASAGQIGGIDRPLFPSFCVPSNE 102

Ta-D --MRKSGG-----GGEDKVMGPLFPRLHVNDTTLKGGGPRAPPRNKMALYEQFSVPSQRFAA---NT-----APAAAHRPAASYAAVS--------SASAGQIGGIDRPLFPSFCVPSNE 97

Hv --MRRAGGGGGGGSGEDKVMGPLFPRLHVNDTTLKGGGPRAPPRNKMALYEQFSVPSQRFAAN---------TAPAAHRPAASFAAVS--------SASAGQIGGIDRPLFPSFCVPSNE 101

Bd --MRRGGGGPAPAGKEDKVMGPLFPRLHVNDT-LKGG-PRAPPRNKMALYEQFSVPSQRFTP---------------HR--ASSSALS--------SASPGQIGGSDRPLFPSFCVPSNE 91

Sb --MRRGGAKDDA--APDKVMGPLFPRLHVNDT-LKGG-PRAPPRNKMALYEQFSVPSHRYSAAAAAPPPPSAPAPSPAPPWRAQRPVP--------ATSASQVGGSDRPLFPSFCVPSTE 106

Si --MRRGAGKDE---APDKVMGPLFPRLHVNDT-VKGG-PRAPPRNKMALYEQFSVPSHRFSAAAAAP-----AAPAPAPPWHAHRPAPGAATSAVPSTSASQAGGSDRPLFPSYCVPSTE 108

Block IV

Os MATRGGGGGGGGKEAKGKVMGPLFPRLHVNDA-AKGGGPRAPPRNKMALYEQFTVPSHRFSGGGGGG------GVGGSPAHSTSAASQS--------QSQSQVYGRDSSLFQPFNVPSNR 105

Zm -MTRGGGGQGG-KEEPGKVMGPLFPRLHVSDA-GKGGGPRAPPRNKMALYEQFTVPSNRFSSPAASA------RAAGASLVPSTAAAQ--------------VYGYDRTLFQPFDVPSNE 97

At --MKRGKDEE-------KILEPMFPRLHVNDA--DKGGPRAPPRNKMALYEQLSIPSQRFGDHGTMN------------------------------------SRSNNTSTLVHPGPSSQ 73

TmDV PVRLPEHIKTNSSGRDG---HATSGRLSTLLKSKDAYAAGSTAECSSSQRRDNNSNNNNNTKNSSGKKLTHDDDFTVPSVFCSGVRPRSNHEEVRIQENSTPFPATSPYKSGPTLSKPTA 219

TmG3 PVRLPEHIKTNSSGRDG---HATSGRLSTLLKSKDAYAAGSTAECSSSQRRDNNSNNNNNTKNSSGKKLTHDDDFTVPSVFCSGVRPRSNHEEVRIQENSTPFPATSPYKSGPTLSKPTA 219

Tu-A PVRLPEHIKTNSSGRDG---HATSGRLSTLLKSKDAYAAGSTAECSSSQRRDNNSNNNNNTKNSSGKKLTHDDDFTVPSVFCSGVRPRSNHEEVRIQENSTPFPATSPYKSGPTLSKPTA 219

Tt-A PVRLPEHIKTNSSGRDG---HATSGRLSTLLKSKDAYAAGSTAECSSSQRRDNNSNNNNNTKNSSGKKLTHDDDFTVPSVFCSGVRPRSNHEEVRIQENSTPFPATSPYKSGPTLSKPTA 219

Tt-B PVRLPEHIKTNSSGRDG---QAISGRLSTQLKSKDAYAAGSTAECSSSQRRDNN---NNSMKNSSGKKLTNDDDFTVPSVFCSGARPRSNHEEVRIQENATPFPATSPYKSGPTVSKPTA 216

Ta-D PVRLPEHIKTNSSGRDG---HATSGRLSTQLKSKDAYAAGSTAECSSSQRRDNNSN-NNNTKNSSGKKLTNDDDFTVPSVFCSGVRPRSNHEEVRIQENSTPFPATSPYKSGPTVSKPTA 213

Hv PVPLPQHINTNSSG------HATSGRLSTQLKSKDAYAAGSTAECTSSHGRDNN------AKNSSGNKLTNDDDFTVPSVFCSGVRPRSNHEEARIQENSTHLPATSPYKSGPTVSKPTA 209

Bd PARSSEHINTNSNGRDGNATRVESGRHSTQLKSKDTYAAGSTAECSS-QRRENS------VKNSSGKKLTNDDDFTVPSVFCSGVPPHSTQEVVRIQEKSTAFPSTSPYKSGPTMSKSSA 204

Sb PVRSSDQMNANSNGRAANGTRAESGRQSTHLKSKDTNAAGPTAECSSRQRENGN-------KNSSGKKLANDDDFTVPSVLYSGVPPHS------SQEKLTLFPTTSPCKSVPAKYSSNG 213

Si PVRSSDHMNANSNGRAGNATRTESGRLSTHLKSKDTNAAGLTAECSSKHRENTT-------KNSSGKKLTNDDDFTVPSVLYSGIPPHS------TQEKFTPFPTKSPYKSMPAMYKSSA 215

Os PGHSTEKINSDKINKKISGSRKELGMLSSQTKGMDIYASRSTAEAP-QRRAENT------IKSSSGKRLADDDEFMVPSVFNSRFPQYSTQENAGVQDQSTPLVAANPHKSPSTVSKSST 218

Zm PPRSSEKFKGNTINGQSNSTRREPLRMSSQTKNKDVCASKSIAKCTSQHRVGNT------IMSS-GKKVVSDDEFMVPSICYPRFYRQSTQDHA---DKSKPQSTTNPHKSP-AMSKSSV 206

At PCGVERNLSVQHLDSSA----------ANQATEKFVSQMSFMENVRSSAQHDQR------------KMVREEEDFAVPVYINS-----------------RRSQSHGRTKSGIEKEKHTP 154

TmDV KFPNTDKRYLEGRNASDTRSMDSPSIIRDKAPANTTTNFLEAEERTSSFQFSAEKTMGKRDDKGSSYSRVKETSSINVSDKQHSRNEGHQARTRNENAAESQNAPKAGNGPYST---DIA 336

TmG3 KFPNTDKRYLEGRNASDTRSMDSPSIIRDKAPANTTTNFLEAEERTSSFQFSAEKTMGKRDDKGSSYSRVKETSSINVSDKQHSRNEGHQARTRNENAAESQNAPKAGNGPYST---DIA 336

Tu-A KFPNTDKRYLEGRNASDTRSMDSPSIIRDKAPANTTTNFLEAEERTSSFQFSAEKTMGKRDDKGSSYSRVKETSSINVSDKQHSRNEGHQARTRNENAAESQNAPKAGNGPYST---DIA 336

Tt-A KFPNTDKRYLEGRNASDTRSMDSPSIIRDKAPANTTTNFLEAEERTSSFQFPAEKTMGKRDDKGSSYSRVKETSSINVSDKQHSRNEGHQARTRNENAAESQNAPKAGNGPYST---DIA 336

Tt-B KFPNTDKRYLEGRNASDTRSRDSPSIIRDKAPANTTTNFLETEERTSSFQFSAEKTMGKRDDKGSSYSRVKETSSINVSDKQHSRNEGHQARTRNENAAESQNAAKAGNGPYST---DVA 333

Ta-D KFPNTDKRYLEGRNASDTRAMDSPSIIRDKAPANTTTNFLEAEERTSSFQFSAEKTMGKRDDKGSSYSRVKETSSINVSDKQHSRNEGHQARTRNENAAESQNAPKAGNGPYST---DVA 330

Hv KFPNTDKRYLEGRNPSDTRSRDSPNIIRDKAPANTTTNFLEAEERTSSFQFSADKTMGKRDDKGSSYR--DKPSSINVSDKQQSRNEGHQARTRNENAAESQNAPKAANGPYST---DIA 324

Bd KCSNTDKRYLEGTNVSDMRSRDSPS-IKDKAPLKTMTN-LDVEERSSSFQISKEK-AGKADDKISSHR--DKLSDLNVFDKQHARTEVHQARRTNENAAESQNAPKAGNGPSST---NVE 316

Sb KR------HLEGIDVSDVKSKGRSG-IKDTEPVQVRID-LEDEETTPSFQILKDK-TGRPDPKVSPFM--DRLKKYNVADKQYSEAESYQMRTRNEDAVKTQNPPKNGSVLLSKPYDDRE 322

Si KCSNTDRTHLEGMKVSDAISMGSPG-IKEKEPTKVRID-LEIEERTSSFQTSKEK-SGRLDPKVSSYR--DKLNKYNVADKQSSEIASYQTRNRKENAGETQNPPEAEMAPSAKPYAGME 330

Os KCYNTVSKKLERIHVSDVKSRTPLK-DKEMEAAQTSKN-VEVE-KSSSFHASKDMFESRHAK---VYPKMDKTGIINDSDEPHGGNSGHQATSRNGGSMKFQNPPMRRNEISSNPSS--E 330

Zm ECYSTVNKHLDKINEADRRLMNSPK-VKEKEAVQGSKA-VEVKEKSSSFQAS-EKFKDKYAK---LCQMRNKASNINHCD-----NNGCQPASVNGNFTEAKNPTAARNTSSCKPCTDVD 315

At MVAPSSHHSIRFQEVNQTGSKQNVCLATCSKPE--VRDQVKANARSGGFVISLDVSVTEEIDLEKSASSHDRVNDYNASLRQESRNRLYRDGGKTR-LKDTDNGAESHLATENHSQEGHG 271

Block II

TmDV CNGA-SKLSEKGLRETGEKRKRST---------------GHHD**L**QR-DDSSDSSVESLPDLEISPDDVVGAIGPKHFWKARRAIVNQQRVFAVQVFELHRLIKVQKLIAASPHLLIEGDP 439

TmG3 CNGA-SKLSEKGLRETGEKRKRST---------------GHHD**V**QR-DDSSDSSVESLPDLEISPDDVVGAIGPKHFWKARRAIVNQQRVFAVQVFELHRLIKVQKLIAASPHLLIEGDP 439

Tu-A CNGA-SKLSEKGLRETGEKRKRST---------------GHHDVQR-DDSSDSSVESLPDLEISPDDVVGAIGPKHFWKARRAIVNQQRVFAVQVFELHRLIKVQKLIAASPHLLIEGDP 439

Tt-A CNGA-SKLSEKGLRETGEKRKRST---------------GHHDVQR-DDSSDSSVESLPDLEISPDDVVGAIGPKHFWKARRAIVNQQRVFAVQVFELHRLIKVQKLIAASPHLLIEWDP 439

Tt-B CNGA-SNLSEKGLRETGEKRKRST---------------GHHDVQR-DDSSDSSVESLPDLEISPDDVVGAIGPKHFWKARRAIVNQQRVFAVQVFELHRLIKVQKLIAASPHLLIEGDP 436

Ta-D CNGA-SNLSEKGLRETGEKRKRST---------------GHHDAQR-DDSSDSSVESLPDLEISPDDVVGAIGPKHFWKARRAIVNQQRVFAVQVFELHRLIKVQKLIAASPHLLIEGDP 433

Hv CNGA-SNLSEKGLRETGEKRKRST---------------GHHDVQR-DDSSDSSVESLPELEISPDDVVGAIGPKHFWKARRAIVNQQRVFAVQVFELHRLIKVQKLIAASPHLLIEGDP 427

Bd RNGA-SNLLEKGLRVTGEKRKRSE---------------GHHNVQK-DDSSDLSVESLPGLEISPDDVVGAIGPKHFWKARRAIVNQQRVFAVQVFELHRLIKVQKLIAASPHLLIEGDP 419

Sb QNGD-SDILKHGLRDTGEKRKRS-----------------HHGVEQNDDLSDSSVEFLAGMEISPDDVVGAIGPKHFWKARRAIVNQQRVFAVQVFELHRLIKVQKLIAASPHLLIEGDP 424

Si QNGN-SDLLELGLRETGEKRKRS-----------------HHGVEHNDDLSDSSVESLPEMEISPDDVVSAIGPKHFWKARRAIVNQQRVFAVQVFELHRLIKVQKLIAASPHLLIEGDP 432

Os NTDRHYNLPQGGIEETGTKRKRLLEQHDAEKSDDVSRLLEQHDAENIDDVSDSSVECITGWEISPDKIVGAIGTKHFWKARRAIMNQQRVFAVQVFELHKLVKVQKLIAASPHVLIESDP 450

Zm SSNRKSNLLERSPREVGAKRKR-----------------GHHNGEQNDDLSDSSVECIPGGEISPDEIVAAIGPKHFWKARRAIQNQQRVFAVQVFELHKLIKVQKLIAASPHLLIEGDP 418

At SPEDIDNDREYSKSRACASLQQIN------------------EEASDDVSDDSMVDSISSIDVSPDDVVGILGQKRFWRARKAIANQQRVFAVQLFELHRLIKVQKLIAASPDLLLDEIS 373

Block III

TmDV CLGSALVTSKKKTAAANVEKQLLSAKSKDDDDAQLTLQQAEYSKDNTEGNQASPSQDNDVVEVRHENQAASNGAVSSNPPAMPAPPDNKQNNW--CAPPPQNQWLVPVMSPSEGFVYKPY 557

TmG3 CLGSALVTSKKKTAAANVEKQLLSAKSKDDDDAQLTLQQAEYSKDNTEGNQASPSQDNDVVEVRHENQAASNGAVSSNPPAMPAPPDNKQNNW--CAPPPQNQWLVPVMSPSEGFVYKPY 557

Tu-A CLGSALVTSKKKTAAANVEKQLLSAKSKDDDDAQLTLQQAEYSKDNTEGNQASPSQDNDVVEVRHENQAASNGAVSSNPPAMPAPPDNKQNNW--CAPPPQNQWLVPVMSPSEGFVYKPY 557

Tt-A CLGSALVTSKKKTAAANVEKQLLSAKSKDDDDAQLTLQQAEYSKDNTEGNQASPSQDNDVVEVRHENQAASNGAVSSNPPAMPAPPDNKQNNW--CAPPPQNQWLVPVMSPSEGFVYKPY 557

Tt-B CLGSALVTSKKKTAAANVEKQLLSAKSKDDDDAQLTLQQAEYSKDNTEGNQASPSQDNDVVEVRHENQAASNGAVSSNPPAMPAPPDNKQNNW--CAPPPQNQWLVPVMSPSEGFVYKPY 554

Ta-D CLGSALVTSKKKTAAANVEKQLLSAKSKDDDDAQLTLQQAEYSKDNTEGNQASRSQDNDVVEVRHENQAASNGAVSSNPPAMPAPPDNKQNNW--CAPPPQNQWLVPVMSPSEGFVYKPY 551

Hv CLGSALVTSKKKTAAANVEKQLLSAKSKDDDDAQLTLQQVEYSKDNTEGNQASPSQDNDLVEVRHENQAASNGAVSSNPPAMPAPTDNKQNNW--CAPPPQNQWLVPVMSPSEGLVYKPY 545

Bd CLGSALATSKKKLAAGNVEKQPPSAKNKDD--AQLTLQQVEYSKDNIEGNQASPSQD-DVVVVQHNNQAASNGGDTSNPPAIPAAPDNKQSNW--CTPP-QNQWLVPVMSPSEGLVYKPY 533

Sb CLGKSLAASKKKLAG-DVEKQLQSAKNNDE-VQPTQQQQLEHSKENTEANQPSPSQD-DAAGVQHNNQAAINGAVSSNPPSMPTPSDNKQNSW--CIPPPPSQWLVPVMSPSEGLVYKPY 539

Si CLDKALAASKKKLAGGDAEKQHQSAKYKDD-VQQT-LQQLEHSKDNTEADQPSPTQD-DVVAVQHNNQAAATAAVNSNPPTMPTPSDNKQNSW--CIPPPPNQWLVPVMSPSEGLVYKPY 547

Os CLGNALLGSKNKLVEENLKAQPLLVATIDD--VEPSLQQPEVSKENTE-DSPPSPHD-TGLGSGQRDQAATNGVSKSNRRATPVASDNKQNNWGVQLQPPQNQWLVPVMSPLEGLVYKPY 566

Zm VLGNALTGKRNKLPKGNSKVQTLSITNKDD--IQPTLEQPELSKQDTEGNLLAHSHD-DGLGDNHHNQAATNETFTSNPPAMHVAPDNKQNNW--CMNPPQNQWLVPVMSPSEGLVYKPF 533

At FLGKVSAKS------YPVKKLLPSEFLVKPPLPHVVVKQRGDSEKTDQHKMESSAENVVGRLSNQGHHQQSNYMPFANNPPASPAPNGYCFPPQPPPSGNHQQWLIPVMSPSEGLIYKPH 487

TmDV TGPCPPAGSILAP-FYASCAPLSLPSTAGEFMNSPYGIPMPHQPQHMGVGGPPA-MPPMYFPPFSVPVMNPVVSSSAVEQVSRVAAARPNTHVEHHSRSSCN---MRNEAVSAGGVWRFH 672

TmG3 TGPCPPAGSILAP-FYASCAPLSLPSTAGEFMNSPYGIPMPHQPQHMGVGGPPA-MPPMYFPPFSVPVMNPVVSSSAVEQVSRVAAARPNTHVEHHSRSSCN---MRNEAVSAGGVWRFH 672

Tu-A TGPCPPAGSILAP-FYASCAPLSLPSTAGEFMNSPYGIPMPHQPQHMGVGGPPA-MPPMYFPPFSVPVMNPVVSSSAVEQVSRVAAARPNTHVEHHSRSSCN---MRNDAVSAGGVWRFH 672

Tt-A TGPCPPAGSILAP-FYASCAPLSLPSTAGEFMNSPYGIPMPHQPQHMGVGGPPA-MPPMYFPPFSVPVMNPVVSSSAVEQVSRVAAARPNTHVEHHSRSSCN---MRNEAVSAGGVWRFH 672

Tt-B TGPCPPAGSILAP-FYASCAPLSLPSTAGEFMNSPYGIPMPHQPQHMGVGGPPA-MPPMYFPPFSVPVMNPVVSSSAVEQVSRVATARPNTHVEHHSRSSCN---MRNEAVSAGGVWRFH 669

Ta-D TGPCPPAGSILAP-FYASCAPLSLPSTAGEFMNSPYGIPMPHQPHHMGVGGPPA-MPPMYFPPFSVPVMNPVVSSSAVEQVSRVAAARPNTHIEHHSRSSCD---MRNEAVSAGGVWRFH 666

Hv TGPCPPAGSFLAP-FYASCAPLSLPSTAGEFMNSPYGIPMPHQPQHMGVGGPPA-MPPMYFPPFSVPVMNPVVSSSAVEQVSRVAAARPNTHLEHHSRSSCN---MRNEAVSVGGVWRFH 660

Bd TGPCPPAGSFLAP-FYASCAPLSLPSTAGDFMNSPYGIHMPHQPQHMGLGGPPP-MPPMYFPPFSMPVMNPVVSASAVEQVSRIAPARPNAHVEHYSRNSCN---MRNEAMSAG-IWRFH 647

Sb SGHCPPAGSFMAPPFFASCGPVSLPSTAGDFMNSAYGVAMPHQPQHMGVPGPPP-MPPMYFPPFSMPVMNPAVSASAVEQVSHVAASQRNGHIEQHTRNSCNASHLRSEAVSAG-VWRVH 657

Si AGHCPPAGSFLAP-FYPSCAPVSLPSTAGDFMSSPYGIPMPHQPQHMGVPGPPPPMPPMYFPPFSMPVMNTAVSASAVEQVSHVAASRPNGHIEQHSRSSCNMSNLRSEALSAD-IWRFH 665

Os SGPCPPAGSILAP-FYANCTPLSLPSTAGDFMNSAYGVPMPHQPQHMGAPGPPS-MPMNYFPPFSIPVMNPTAPAPVVEQGRHPSMPQPYGNFEQQSWISCN-------MSHPSGIWRFH 677

Zm AGPCPPVGNLLTP-FYANCAPSRLPSTP-------YGVPIPHQPQHMVPPGAPA-MHMNYFPPFSMPVMNPGTPASAVEQGSHAAAPQPHGHMDQQSLISCN-------MSHPSGVWRFL 637

At PGMAHTG----------HYGGYYGHYMPTPMVMPQYHPGMGFPPPGNGYFPPYGMMPTIMNPYCSSQQQQQQQPNEQMNQFGHPGNLQNTQQQQQRSDNEPAP------QQQQQPTKSYP 591

Block VI

TmDV SSRGSELQ**R**SSAASSPFDRQQGQGEAR**G**PAAAAPAAPLPTSS------------------------AGN**G**NAAQQPQVSSGSQENPVAAAA--RVIRVVPHTARTASESAARIFRSIQME 766

TmG3 SSRGSELQ**G**SSAASSPFDRQQGQGEAR**D**PAAAAPAAPLPTSS------------------------AGN**A**NAAQQPQVSSGSQENPVAAAA--RVIRVVPHTARTASESAARIFRSIQME 766

Tu-A SSRGSELQGSSAASSPFDRQQGQGEARGPAAAAPAAPLPTSS------------------------AGNGNAAQQPQVSSGSQENPVAAAA--RVIRVVPHTARTASESAARIFRSIQME 766

Tt-A SSRGSELQGSSAASSPFDRQQGQGEARGPAAAAPAAPLPTSS------------------------AGNGNAAQQPQVSSGSQENPVAAAA--RVIRVVPHTARTASESAARIFRSIQME 766

Tt-B SSRGSELQGSSAASSPFDRQQGQGEARGPAAAAPAAPLPTSS------------------------AGNGNAAQQPQVSSSSQENPVAAAA--RVIRVVPHTARTASESAARIFRSIQME 763

Ta-D SSRGSELQGSSAASSPCDRQQGQGEARGPAAAAPAAPLPTSS------------------------AGNGNAAQQPQVSSGSQENPVAAAA--RVIRVVPHTARTASESAARIFRSIQME 760

Hv SSRGSELQWSSAASSPFDRQQGQGEARGHAAAAPAAPLPTSSSA---------------------GNGNGNAAQQPQVSSGSQENPVAAAAAARVIRVVPHTARTASESAARIFRSIQME 759

Bd ASRDSELQASSAASSPFDRQQG--EARGPAAPP---PIPTSS------------------------AGNG----QPQPSTGSKENP---AG---VIRVVPHTARTASESAARIFRSIQME 728

Sb ASRDSELQGS-SASSPFDRQQG--EGRG-----PAPPFPASS------------------------VGNRQAQAQAQASSGSRENPS------RVIRVVPHTARTASESAARIFQSIQME 739

Si ASKDSELQGS-SASSTFDRQQG--EGRG-----PAQPFPSSS------------------------VGN----GQPQPSSGSRENPG------RVIRVVPHTSRTASESAARIFESIKME 743

Os ASRDSEAQAS-SASSPFDRFQCSGSG--PVSAFPTVSAQNNQP---------------------------------QPSYSSRDNQT------NVIKVVPHNSRTASESAARIFRSIQME 755

Zm ASRDSEPQAS-SATSPFDRLQVQGDGSAPLSFFPTASAPNVQPPPSSGGRDRDQQNHVIRVVPRNAQTASVPKAQPQPSSGGRDQKN------HVIRVVPHNAQTASESAAWIFRSIQME 750

At RARKSRQGSTGSSPSGPQGISGSKSFRPFAAVDEDSNINNAP-------------------------EQTMTTTTTTTRTTVTQTTRDGGGVTRVIKVVPHNAKLASENAARIFQSIQEE 686

TmDV RQQNGP--- 772

TmG3 RQQNGP--- 772

Tu-A RQQNGP--- 772

Tt-A RQQNGP--- 772

Tt-B RQQNGP--- 769

Ta-D RQQNGP--- 766

Hv RQQNGP--- 765

Bd RQQNDP--- 734

Sb RKQNDP--- 745

Si RQQND---- 748

Os RQRDD---- 760

Zm RNQNDS--- 756

At RKRYDSSKP 695

**Fig. S2** GRIK1-like protein alignment. Conserved Serine/Threonine protein kinase domain is indicated in red. Black shading indicates 100% conservation. Polymorphic amino acids between DV92 carrying the late flowering *Eps-A^m^1-l* allele and G3116 carrying the early flowering *Eps-A^m^1-e* allele are highlighted in yellow. Species abbreviations and sequence IDs: *Triticum monococcum* accession DV92, TmDV (AC270217.1); *Triticum monococcum* accession G3116, TmG3 (KU570057); *Triticum urartu*, Tu-A (UCW_Tu-k35_contig_23725); *Triticum turgidum* ssp. *durum* A genome, Tt-A (Td-k56_contig_27681); *Triticum turgidum* ssp. *durum* B genome, Tt-B (Td-k36_contig_30016); *Hordeum vulgare,* Hv (BAJ87365.1); *Brachypodium distachyon,* Bd (Bradi1g11340.1); *Sorghum bicolor,* Sb (XP_002446858.1); *Setaria italica,* Si (XP_004976369.1); *Oryza sativa,* Os (LOC_Os03g50330.1); *Zea mays*, Zm (XP_008669092.1); *Arabidopsis thaliana,* At (AT5G60550.1)

TmDV MS----CCCCSCFGFLSKLHH-RPAAGDSDGAPSKDLLLPRSSD--GGSFYAGD------------NSSSFLGDESRS----FYEREEEDYLLRQSDGD--**D**EPPRKRSEDIILSRARNG 95

TmG3 MS----CCCCSCFGFLSKLHH-RPAAGDSDGAPSKDLLLPRSSD--GGSFYAGD------------NSSSFLGDESRS----FYEREEEDYLLRQSDGD--**E**EPPRKRSEDIILSRARNG 95

Tu-A MS----CCCCSCFGFLSKLHH-RPAAGDSDGAPSKVLLLPRSSD--GGSFYAGD------------NSSSFLGDESRS----FYEREEEDYLLRQSDGD--DEPPRKRSEDIILSRARNG 95

Tt-A MS----CCCCSCFGFLSKLHH-RPAAGDSDGAPSKVLLLPRSSD--GGSFYAGD------------NSSSFLGDESRS----FYEREEEDYLLRQSDGD--DEPPRKRSEDIILSRARNG 95

Tt-B MAG--CCCCCSCFGFLWKQPH-RPAAGDSDGAPSKDLLLPRSND--GGSFYAGDDPD---------NSSSFLGDDSRS----FYEREEEDYLLRQSDGDGDDKPPRKRSEDIILSRARNG 102

Hv MAADMAGCCYSCFGFLRKHHR-RRRR-----PPSKDLLLPRSSDDDGSGFYPGDDPG---------NSSSFLGDDSRSRSRSFCEREEEEYLLR-DDGD--GEPPRKRSEDIILSRARNG 102

Bd MADLTDLGCCSCFSFLRKPSVKVGRPRETDGILSQDLLKRQSSEDLDGSFYTGDDPDRSFYNGNDLDRSFYNGDDPDR---SFYDRDGTDYVHESDDEP-----PRKRSEDIILSRAQSG 112

Sb MADITDIGCCSCFSFLRKPSVPARQHQDADAMLSEDLLKRQSAEDPDGSFYTGDDPDISFYNGDDLDRSFYNGDDPDR---SFYDRDDTDYLEGSDDGP-----PRKSSEDIIQSRTQNG 112

Si MADLTDIGCCSCFSFLRKPSVPARQPREADGILSEDLLKRQSAEDPDGSFYTGDDPDVSFYNGDDLDRSFYNGDDPDR---SFYDRDDAEYLHGSDDGP-----PRKTSEDIIQSRAQNG 112

Os MADLTDMGCCSCFGFLRKPRVSVSRPRDADGILSEDLLNHKSAEDPDGSFYTGDDPDRSFYDRDDLDRSFYNGDDPDR---SFYDGDDPDHLYGSDDGQ-----PRKRSEDIILSRAQNG 112

Zm MADLTDIGCCCCFSFLRKPSVPVCEHQDADGMLTEDLLKRQSAEDHDGSFYTGDDP------GDDLDRSFYNGDDHDR---SFYDRDDSDYLDGSDDGP-----SRKSSEDIIQLRAQNG 106

At --------------MFRDSFLFARTIGCFGCFGSSGSRNQQSPKPYDDDTHSCDS-----------DVTSTARGE---------EEEDEEEVEQKSRSK--------RSEEILKYRLDNG 78

Serine/Threonine protein kinase

:: . :. :* . ... :. *

TmDV FACRDSLVRDTRKLFRSEDETTGCKMINQYVHLGKIGAGSYGKVVKYRNIKDGRLYAIKVLSKPYMLKVRVVRSETAMTDVLREVSLMKMLDHPNIVNLIEVIDDPNTDKFYMVLEYVEG 215

TmG3 FACRDSLVRDTRKLFRSEDETTGCKMINQYVHLGKIGAGSYGKVVKYRNIKDGRLYAIKVLSKPYMLKVRVVRSETAMTDVLREVSLMKMLDHPNIVNLIEVIDDPNTDKFYMVLEYVEG 215

Tu-A FACRDSLVRDTRKLFRSEDETTGCKMINQYVHLGKIGAGSYGKVVKYRNIKDGRLYAIKVLSKPYMLKVRVVRSETAMTDVLREVSLMKMLDHPNIVNLIEVIDDPNTDKFYMVLEYVEG 215

Tt-A FACRDSLVRDTRKLFRSEDETTGCKMINQYVHLGKIGAGSYGKVVKYRNIKDGRLYAIKVLSKPYMLKVRVVQSETAMTDVLREVSLMKMLDHPNIVNLIEVIDDPNTDKFYMVLEYVEG 215

Tt-B FACRDSLVRDTRKLFRSEDETTGCKMINQYVHLGKIGAGSYGKVVKYQNIKDGRLYAIKVFSKPYMLKVRVVRSETAMTDVLREVSLMKMLDHPNILNLIEVIDDPNTDKFYMVLEFVEG 222

Hv FACRDGLVRDTRRLFRSEDETTGCKMINQYVHLGKIGAGSYGKVVKYRNIKDGRLYAIKVLSKPYMLKVRVVRSETAMTDVLREVSLMKMLDHPNIVNLIEVIDDPNTDKFYMVLEYVEG 222

Bd FACRESLVKETKKVFRSEDEL-GNKMINQYVHLGKIGAGSYGKVVLYRNIKDGKLYAVKVLNKPYMMKVRVVRSETAMSDVLREVSLMKMLDHPNVVNLIEVIDDPNTDKFYMVLEYVEG 231

Sb FACREIPVKETKKVFRSEDEN-GNKMVNQYVHLGKIGSGSYGKVVLYRNIKDGKLYAVKVLNKPYMMKVRVVRTETAMTDVLREVSIMKMLNHPNIVNLVEVIDDPNIDKFYMVLEYVEG 231

Si FACREIPVKETKKVFRSEDEN-GYKMVNQYVHLGKIGSGSYGKVVLYRNIKDGKLYAVKVLNKPYMMKIRVVRSETAMTDVLREVSIMKMLNHPNIVNLVEVIDDPNIDKFYMVLEYVEG 231

Os FACRESLVKETKKVFRSEDEN-GSKMVNQYVHLGKIGSGSYGKVVLYRSMKDGKLYAVKVLNKSYMMKVRVVRSETAMTDVLREVSIMKMLDHPNIVNLIEVIDDPNADKFYMVLEYVEG 231

Zm FACREVPVKETKKVFRSEDEN-GNKMVNQYVHLGKIGSGSYGKVVLYRNIKDGKLYAVKVLNKPYMMKVHVVHTKTAMTDVLREVSIMKMLNHPNIVNLVEVIDDPNMDKFYMVLEYVEG 225

At LICRHIPVRETNELIRGEDEN-GDKTINEYVRVCKIGSGSYGKVVLYRSTLDGQYYAIKAFHKSHLLRLRVAPSETAMSDVLREVMIMKILEHPNIVNLIEVIDDPETDHFYMVLEYVDG 197

Serine/Threonine protein kinase

TmDV KMVCNNGI---GLGE**A**TSRKYLRDIVSGVMYLHSHNIIHGDIKPDNLLVTSTGNVKIGDFSVSQIFEDDDDMLWRSPGTPVFTAPECCQGS--AYHGRTADTWAVGVTLYCMITGKYPFL 330

TmG3 KMVCNNGI---GLGE**S**TSRKYLRDIVSGVMYLHSHNIIHGDIKPDNLLVTSTGNVKIGDFSVSQIFEDDDDMLWRSPGTPVFTAPECCQGS--AYHGRTADTWAVGVTLYCMITGKYPFL 330

Tu-A KMVCNNGI---GLGEATSRKYLRDIVSGVMYLHSHNIIHGDIKPDNLLVTSTGNVKIGDFSVSQIFEDDDDMLWRSPGTPVFTAPECCQGS--AYHGRTADTWAVGVTLYCMITGKYPFL 330

Tt-A KMVCNNGI---GLGEATSRKYLRDIVSGVMYLHSHNIIHGDIKPDNLLVTSTGNVKIGDFSVSQIFEDDDDMLWRSPGTPVFTAPECCQGS--AYHGRTADTWAVGVTLYCMITGKYPFL 330

Tt-B KMVCNNGI---GLGEATSRKYLRDIVSGVMYLHSHNIIHGDIKPDNLLVTSTGNVKIGDFSVSQIFEDDDDMLWRSPGTPVFTAPECCQGS--AYHGRTADTWAVGVTLYCMITGKYPFL 337

Hv KMVCDNGI---GLGEATSRKYLRDIVSGVMYLHSHNIIHGDIKPDNLLVTSTGNVKIGDFSVSQIFEDDDDMLWRSPGTPVFTAPECCQGS--AYHGRAADTWAVGVTLYCMITGKYPFL 337

Bd KMVCDN-----GLGEATSRKYLRDIIAGVIYLHSHNIIHGDIKPDNLLVTSTGNVKIGDFSVSQIFEDDDDLLWRSPGTPVFTAPECCQGS--AYHGRAADTWAVGVTLYCMITGHYPFL 344

Sb KMVCDN-----GLEEATARNYLRDIISGLMYLHSHNVIHGDIKPDNLLVTSAGNVKIGDFSVSQVFEDDDDMLWRSPGTPVFTAPECCQGS--AYHGRASDTWAVGVTLYCMVSGHYPFL 344

Si KMVCDN-----GLEEATARNYLRDIISGLMYLHSHNVIHGDIKPDNLLVTSTGNVKIGDFSVSQVFEDDDDMLWRSPGTPVFTAPECCQGS--AYHGRASDTWAVGVTLYCMISGQYPFL 344

Os KMVCDN-----GLGEATSRNYLRDIISGVMYLHSHNIIHGDIKPDNLLVTSTGSVKIGDFSVSQIFEDDDDLLWRSPGTPVFTAPECCQGS--AYHGRAADTWAVGVTLYCMITGHYPFL 344

Zm KMVCDN-----GLEEATARFFLRDIISGLLYLHSHNVIHGDIKPDNLLVTSTGNVKIGDFSVSQVFEDDDDMLWRSPGTPVFTAPECCQGS--AYHGRASDTWAVGVTLYCMVSGHYPFL 338

At KWVYDGSGPPGALGEKTARKYLRDIVTGLMYLHAHDVIHGDIKPDNLLVTSSGTVKIGDFSVSQVFKDDDDQLRRSPGTPVFTAPECCLVSGITYSGRAADTWAVGVTLYCMILGQYPFL 317

Serine/Threonine protein kinase

TmDV GETLQETYDKIANDPVEIPG**D**TSPQLADLMQRLLYKDPGDRMTLQAVAAHPWVAGAEGPVPEFVCRCGFGRRNRSDSQEAVQ-------- 412

TmG3 GETLQETYDKIANDPVEIPG**N**TSPQLADLMQRLLYKDPGDRMTLQAVAAHPWVAGAEGPVPEFVCRCGFGRRNRSDSQEAVQ-------- 412

Tu-A GETLQETYDKIANDPVEIPGDTSPQLADLMQRLLYKDPGDRMTLQAVAAHPWVAGAEGPVPEFVCRCGFGRRNRSDSQEAVQ-------- 412

Tt-A GETLQETYDKIANDPVEIPGDTSPQLADLMQRLLYKDPGDRMTLQAVAAHPWVAGAEGPVPEFVCRCGFGRRNRSDSQEAVQ-------- 412

Tt-B GETLQETYDKIANDPVEIPGDTSPQLADLMQRLLYKDPGDRMTLQAAAAHPWVAGAEGPVPEFVCRCGFGRRNRNDSQEAVQ-------- 419

Hv GETLQETYDKIANDPVEIPGDTSPQLADLMQRLLYKDPGDRMTLQAAAAHPWVAGAEGPVPEFVCRCGFGRRDRNDSQEAVQ-------- 419

Bd GDTLQETYDKIVNDPVEIPDDMNPQLADLLQKILCKDPGDRITLLAAAEHPWVAGDEGPVREYFCRCGFGRRKRIDFQETVQ-------- 426

Sb GDTLQETYDKIANDPVQIPGDMNPQLADLLLRLLCKDPGDRITLQAAAEHPWVAGGEGPVPEFICRCGFGRRKRNDVREEVQ-------- 426

Si GDTLQETYDKIANDPVQIPDDMNPQLADLLQRLLCKDPGDRITLQAAAEHPWVAGDKGPVPEFFCRCGFGRRKRNDFREEVQ-------- 426

Os GDTLQETYDKIVNDPVQIPDNMNPQLADLLERLLCKDPANRITLQAVGEHPWVAGDQGPVVEYFCRCGFGRRKRDDLKGEVQ-------- 426

Zm GDTLQETYDKIANDPVELPGDVNPQLADLLLRLLCKDPGDRITLRAAAEHPWVAGDEGPVPEFICRCGFGRRKRNDVREEVQ-------- 420

At ADTLQDTYDKIVNNPLIIPDGLNPLLRDLIEGLLCKDPSQRMTLKNVSEHPWVIGEDGHVPEYFCWCKRNAASKIEEGEANGISETSDPN 407

**Fig. S3** PWWP1 protein alignment. Conserved PWWP domain is indicated in red. Black shading indicates 100% conservation. Polymorphic amino acids between DV92 carrying the late flowering *Eps-A^m^1-l* allele and G3116 carrying the early flowering *Eps-A^m^1-e* allele are highlighted in yellow. Species abbreviations and sequence IDs: *Triticum monococcum* accession DV92, TmDV (AC270203.1); *Triticum monococcum* accession G3116, TmG3 (KU570056); *Triticum urartu*, Tu-A (UCW_Tu-k41_contig_8163); *Triticum turgidum* ssp. *durum* A genome, Tt-A (UCW_Tt-k31_contig_36721); *Hordeum vulgare,* Hv (BAK01602.1); *Brachypodium distachyon,* Bd (Bradi2g14280); *Sorghum bicolor,* Sb (Sobic.009G257500.1); *Zea mays*, Zm (XP_008656118.1)

PWWP domain

TmDV MGKANGVGVGVGVDADVSAVGALVWVRRPNGSWWPGRVASRLELPDGCPAPPRSPATPILLLGRR-DGPAFVEWCNLERSKRVKPFRCGEADLDDLIRRAEEQAARRRRAS--------- 110

TmG3 MGKANGVGVGVGVDADVSAVGALVWVRRPNGSWWPGRVASRLELPDGCPAPPRSPATPILLLGRR-DGPAFVEWCNLERSKRVKPFRCGEADLDDLIRRAEEQAARRRRAS--------- 110

Tu-A MGKANGVGVGVGVDADVSAVGALVWVRRPNGSWWPGRVASRLELPDGCPAPPRSPATPILLLGRR-DGPAFVEWCNLERSKRVKPFRCGEADLDDLIRRAEEQAARRRRAS--------- 110

Tt-A MGKAN--GVGVGVDADVSAVGALVWVRRPNGSWWPGRVASRLELPDGCPAPPRSPATPILLLGRR-DGPAFVEWCNLERSKRVKPFRCGEADLDDLIRRAEEQAARRRRAS--------- 108

Hv MGKAD-----------VSAVGALVWVRRPNGSWWPGRVASRLELPDDCPAPPRSPATPILLLGRR-DGTAFVEWCNLERSKRVKPFRCGEADLDDLIRRTEEQTARRRRAG--------- 99

Bd MGGLE--AQAAPEVVDVSAAGTLVWVRRPNGSWWPGQVLSRADVPDGCPAPPRSPATPIMLLGRR-DGPAFVDWCNLERSKRVKPFRCGDLDLDDCIRKAQQQQAARRRGSTSTNRRRVC 117

Sb MGALP--KEGAP-AVDVSVEGTLVWLRRPNGSWWPSIVISPQDVPEGCPPPPRCPATPIMLLGRRPDGPTYVDWCNLDRCKRVKPFRCGELDFEQRITNALTLAATGNRSTWSYNK---- 113

Zm MGGLP--KEGAP-AVDVSVEGTLVWLRRPNGSWWPSIVISPQDVPEGCAPPPRCPATPIMLLGRRPDGPTYVDWCNLDRCKRVKPFRCGELDLEQRIANALTLAATSDRSTWSYNK---- 113

TmDV ----AARNARKEDAVLQALDIERARL---------RLRPRAPPPTASCP--------PPPPPP-------------------RKRKTPNDSEDDAP-AARRMRDLTDIGS---PPKPTTG 186

TmG3 ----AARNARKEDAVLQALDIERARL---------RLRPRAPPPTASCP--------PPPPPP**PPPP**---------------RKRKTPNDSEDDAP-AARRMRDLTDIGS---PPKPTTG 190

Tu-A ----AARNARKEDAVLQALDIERARL---------RLRPRAPPPTASCP--------PPPPPPP------------------RKRKTPNDSEDDAP-AARRMRDLTDIGS---PPKPTTG 187

Tt-A ----AARNARKEDAVLQALDIERARL---------RLRPRAPPPTASCP--------PPPPPP-------------------RKRKTPNDSEDDAP-AARRMRDLTDIGS---PPKPTTG 184

Hv ----SSRNARKEDAVLQALDIERARLG---LGLALRLRPRAPSTAAQQG--------PPSGPPPP-----------------RKRKTPNDSEDDAP-AARRMRDLTDIGS---PPKPTTG 183

Bd NTANGARYARKEDAVLQALQIER------ATNNRQGAKSS--RKPSSLP-------AAANPPP----------------LPKRKRRTPNDSEDDVPQGFRRMRDLTEIGSDAAVFPDSTC 206

Sb -----GRYARMEDAILQALDIEKERALGSKTKAYLHAASCSPSPKTEMTNGQVKDATAKDASPTIQPSPLLLPLPLPPLPPKRKRKTPYDSEDDAPKGSRRMRDLRDIGSKTVPPMDLVN 228

Zm -----GRYARMEDAILQALDIEKELALGPATKAYLHARSCSPRPKVEMPNGQVKDTAAKDHAPTIQP-------PLP--PPKRKRKTPYDSEDDVPEVSRRMRDLRDIGSKAVVPMDLAN 219

TmDV QMTRSRQAHHDATAAAAAKRSKVPPTVD------QVDNNLPCGALRKKDRSRPLSELCNG--------------------------VKPSN--GLDT**S**LDK--------FKPEQPKGVNG 264

TmG3 QMTRSRQAHHDATAAAAAKRSKVPPTVD------QVDNNLPCGALRKKDRSRPLSELCNG--------------------------VKPSN--GLDT**W**LDK--------FKPEQPKGVNG 268

Tu-A QMTRSRQAHHDATAAAAAKRSKVPPTVD------QVDNNLPCGALRKKDRSRPLSELCNG--------------------------VKPSN--GLDTSLDK--------FKPEQPKGVNG 265

Tt-A QMTRSRQAHHDATAAAAAKRSKVPPTVD------QVDNNLPCGALRKKDRSRPLSELCNG--------------------------VKPSN--GLDTSLDK--------FKPEQPKGVNG 262

Hv QMTRSRQARHDA------KRSKVPPSTD------QVDNNLPCGLSRKKDRSRPLSELCNGG-------------------------FKPSNGLGLDTSLDK--------LKPEQAKGVNG 258

Bd CLPASAS---------QMKRSRQSHHDSGKRKHPTLDQDQPCGMLRKKDRSRPLSELCNGDMWNGFKPNAQRADHDQQLMRMGTCSGSSSASSSLDTLADKSSSHPTALFKTDQAKG--- 314

Sb AAAISATKYDDLPNDGQVKRIVHSQATT-KRKHADTHQDQPCGIPRKKDRSRPLSELCNGDMWNGSRPNGQKAD--EHLLGVATCSSSSSGTSTLDTPLDTNSCHRSAAFKTDQAKGTE- 344

Zm AAAVSAPKYDDLTNAGQAKRIVHSQATA-KRKHAGLHQDQPCGIPRKKDRSRPLSELCNGDMWGGSRPNGQKAD--EHLLGVATCSSSSSGTSTLDTPLDMNNCHRSMAFKTDQAKGTE- 335

TmDV TSCVPRPLDDAFARGPVDQPVDAPIAAA**M**TILKADHLHAHQPCAPTKAPPTWECTKRASNC**S**KDGMSSQCDSRNPKKKTTSSAGHEGGGRTR--------KGRTAKQKAAPINEVILLKR 376

TmG3 TSCVPRPLDDAFARGPVDQPVDAPIAAA**T**TILKADHLHAHQPCAPTKAPPTWECTKRASNC**I**KDGMSSQCDSRNPKKKTTSSAGHEGGGRTR--------KGRTAKQKAAPINEVILLKR 380

Tu-A TSCVPRPLDDAFAHGPVDQPVDAPIAAATTILKADHLHAHQPCAPTKAPPTWECTKRASNCSKDGMSSQCDSRNPKKKTTSSAGHEGGGRTR--------KGRTAKQKAAPINEVILLKR 377

Tt-A TSCVPRPLDDAFARGPVDQPVDAPIAAATTILKADHLHAHQPCAPTKAPPTWECTKRASNCSKDGMSSQCDSRNPKKKTTSSAGHEGGGRTR--------KGRTAKQKAAPISEVILLKR 374

Hv TSCVTRPLDDAFTRGPG--PGDQP--AAPSISKADHLHAHQPCAATKAPPTWECTKRASNCSKDGMSSQCDSRNPKKKTMSSADHEGGGRTR--------KGRAAKHKPAPSNEVVLLKR 366

Bd ---ITRLPNDDLPRGDE----------LGSIFKADRLHVDQPGALMKDP-SWECSKQDPDSSKADLSSQCDGRNHKKKTISSVDQEGSNRAKSVLEREHCKSRVVKYKAPS-NEGVLLEE 419

Sb ISCMTRLLSDDSRHGNDF---VETPPAAQKILESDHLHRYQPSGLTENP-TWKSNKQIHDSRKAEKRAR---KNIKMRTISSVDQEGKNRTRDSNDRDHHKTKIGKHKVAR-DEVVLLEK 456

Zm ISCMTRLLSDDSRHGNDF---VGTP-AAQNIVEPDHLHRYQPCGLAKNP-TWKPNKQTNDARKAEKHAR---KNMKMRTISSVDQENKNRTRDSNDHDHHKPKTGKHKSPR-DGVVLLEK 446

TmDV RTAKSAAT----PDEDDKSLVVMLPD--ALVHAALRQRSEIKCEPEEPSGTIGRHHSNGETGSVPPVVSEPAPPQRRD------LRCHVAVKPTKTLQLNPSLYDVEIS-SGPGCGGGSK 483

TmG3 RTAKSAAT----PDEDDKSLVVMLPD--ALVHAALRQRSEIKCEPEEPSGTIGRHHSNGETGSVPPVVSEPAPPQRRD------LRCHVAVKPTKTLQLNPSLYDVEIS-SGPGCGGGSK 487

Tu-A RTAKSAAT----PDEDDKSLVVMLPD--ALVHAALRQRSEIKCEPEEPSGTIGRHHSNGETSSVPPVVSEPAPPQRRD------LRCHVAVKPTKTLQLNPSLYDVEIS-SGPGCGGGSK 484

Tt-A RTAKSAAT----PDEDDKSLVVMLPD--ALVHAALRQRSEIKCEPEEPSGTIGRHHSNGETGSVPPVVSEPAPPQRRD------LRCHVAVKPTKTLQLNPSLYDVEIS-SGPGCGGGSK 481

Hv RTAKSAAA----PDEDDKSLVVMLPG--ALVHATLWQRSEIKCEPEEPSGTIGRYHSNGETGSVPPVMSEPAPPQRRD------LRCHVAVKPTKTLQLNPSLYDVELS-SGQGCGGGSK 473

Bd RLERSTVD--KTAAPDDDKDLAILPNDLDRVDAVLQQCSESKHKHEEPSETTS-NNSYCENVSVSSVAFELPPQHTDP-----AVASCHAVKATKTLQLNSALYDVELSVQGSSSSSNNK 531

Sb RLEKHSLN--KPAEHDTKMHIAVVPSGMDCVGAVQQQHCQSKCDPEESLETLS-NHSNCESGSVSSLVFEKPLQVLSPEKKPLGHERCHAVKPIKTLHLNPTLYDVELSVLGSS----NS 569

Zm RQEKRSLDKNKPAEHDIKMHIAVVPSGMDCVGAFQQQRFKSRCDPEESLESLS-NHSNCESGSVSSLVFEKPLQALPPGKNLLGYEICHAAKPIKTLHLNPALYDVELSVLGSS----NT 561

TmDV GRHVPLVSLMSRSSRRPVVGYPVSVEVLDAACCHPPAPSVDGVPDAARRHPPAPSVD**G**VRDAVRCHPPAPSVTGVLDATCRHPPARSIDGVPDAARRHPPAPSVGRVRDAVRRHPPAPGV 603

TmG3 GRHVPLVSLMSRSSRRPVVGYPVSVEVLDAACCHPPAPSVDGVPDAARRHPPAPSVD**C**VRDAVRCHPPAPSVTGVLDATCRHPPARSIDGVPDAARRHPPAPSVGRVRDAVRRHPPAPGV 607

Tu-A GRHVPLVSLMSRSSRRPVVGYPVSVEVLDAACCHPPAPSVDGVPDAARRRPPAPSVDGVRDAVRCHLPAPSVTGVLDATCRHPPARSIDGVPDAARRHPPAPSVGRVRDAVRRHPPAPGV 604

Tt-A GRHVPLVSLMSRSSRRPVVGYPVSVEVLDAACCHPPAPSVDGVPDAARRHPPAPSVDGVRDAVRCHPPAPSVTGVLDATCRHPPARSIDGVPDAARRHPPAPSVGRVRDAVRRHPPAPGV 601

Hv GRHVPLVSLMSRSSRRPVVGYPVSVEVLDATGCHPPAPNVDGVPNAARHQTSAPRVAGVQDAVRRQPPAPSVNGVRDIMRCLPPAPSVNGVPDAARRHPTAPSVDGVRDAAHRHLPTRGV 593

Bd GRHVPLVSLMSRSSRRPVVGYPVSVEVLDVVYCP-------------------------------------------------PASSID------------------------------- 571

Sb GRRVPLVSLMSRCNRKPVVGYPVSVEVSDDVFDR-------------------------------------------------PLSRKD------------------------------- 609

Zm GRRVPLVSLMSRWNCKAVVGYPVSVEVSDDVFGCP-----------------------------------------------SPSSEKD------------------------------- 603

TmDV GGVDHPPSTSSGAHRLVKVKEEVEEEAPQRAVPASAQRARAKRSRRKASEDELWRPHSKEEET-AAPRPVRARRSVRRKAGSEDESWRPHSKKPAVA**A**SAAVSSPRKMRRLSSLGATSQR 722

TmG3 GGVDHPPSTSSGAHRLVKVKEEVEEEAPQRAVPASAQRARAKRSRRKASEDELWRPHSKEEET-AAPRPVRARRSVRRKAGSEDESWRPHSKKPAVA**V**SAAVSSPRKMRRLSSLGATSQR 726

Tu-A GGVDHPPSTSSGAHRLVKVKEEVEEEAPQRAVPASAQRARAKRSRRKASEDELWRPHSKEEET-AAPRPVRVRRSVRRKAGSEDESWRPHSKKPAVAVSAAVSSPRKMRRLSSLGATSQR 723

Tt-A GGVDHPPSTSSGAHRLVKVKEEVEEEAPQRAVPASAQRARAKRSRRKASEDELWRPHSKEEET-AAPRPVRVRRSVRRKAGSEDESWRPHSKKPAVAVSAAVSSPRKMRRLSSLGATSQR 720

Hv SGGDHPPSTSSGANGLVKVKEEVEEEAPQRAVPASAQRVRAKRSRRKASEDELWRPHSKEEEVGAAPRPVRARRSVRRKVASEDELWRPHSKKPAVSVS-AVSSPRKMRRLSSLGARSQR 712

Bd ---DDHPSTSS-ANGLVKEQE---TAVPQCAMPSSHKG-RAKARSR-RKTSED----------------------------DMDKSWRPHNKNP-------VSSPRKMRRLSSFAT-SQR 646

Sb ---VQQPATSS-VNGIVLKKDE-TESLQ-CLVPSSAQTSRPKPKSRSRRASEK----------------------------EVDKLWQPHTKKP-------ASSSRKMRRLSSFAS-GQR 687

Zm ---IQQAATNS-VNGIVLKKDE-TEGLERVVVPPSAQTSRPRCKSRSRRASEK----------------------------AVDKLWQPHTKWP-------ASSSRKMRRLSSFGP-GQR 682

TmDV GGGDEERRRR**RR**KGPGAG**TG**QLVVACVPVRVVFSRIKEALVSQPLKLKSK------------ 772

TmG3 GGGDEERRRR**--**KGPGAG**--**QLVVACVPVRVVFSRIKEALVSQPLKLKSK------------ 772

Tu-A GGDDEERRRRR-KGPGAG--QLVVACVPVRVVFSRIKEALVSQPLKLKSK------------ 770

Tt-A GGGDEERRRR--KGPGAG--QLVVACVPVRVVFSRIKEALVSQPLKLKSK------------ 766

Hv GGGDEERRRR--KGPGAGTGQLVVACVPVRVVFSRIKEALVSQPLKLKSK------------ 760

Bd GGEDRKTLVG--KFCGTA-----VACIPLRVVFSRINEALSYSAK----------------- 684

Sb DGDDRKSAVG--KVAGAT-----IACIPLRVVFSRINEALSFSVNENALAGCGAAPRLRRR- 741

Zm DG---KSAVR--KVSGAT-----VACIPLRVVFSRINEALSFSVNENALAGCGTASRLRNTW 734

**Fig. S4** **a** Introgression of the *Eps-A^m^1* locus from *Triticum monococcum* accession DV92 into *T. turgidum* ssp. *durum* cultivar CBW0112. **b** Schematic representation of the recombinant 1A chromosome carrying the *Eps-A^m^1* locus from DV92. The size of the introgressed 1A^m^ chromosome region is expected to be less than 35 cM, based on the position of *wmc716* (last 1A marker) in Somers et al. (2004).

***Triticum turgidum*** (AABB)

Langdon

***Triticum monococcum*** (A^m^A^m^)

DV92

**BC5**

(2n=28, AABB)

**ABA^m^**

**AABBA^m^A^m^**

**X**

**X**

**CBW0112**

**BC1** (2n=35, AAA^m^BB)

Colchicine

**X**

**CBW0112** (AABB)

*wg241*

*ELF3*

***Eps-A^m^1***

*NDK3*

*barc287*

**1A**

**1A^m^**

*GLU-A3*

*barc263*

*gwm136*

*gwm164*

*gwm135*

*gwm357*

*wmc312*

*wmc716*

**b**

**a**

**Fig. S5** Expression profiles of A-genome homoeologs of *Eps-A^m^1* candidate genes based on published RNAseq data (IWGSC et al. 2014) generated by WheatExp (Pearce et al. 2015)

**
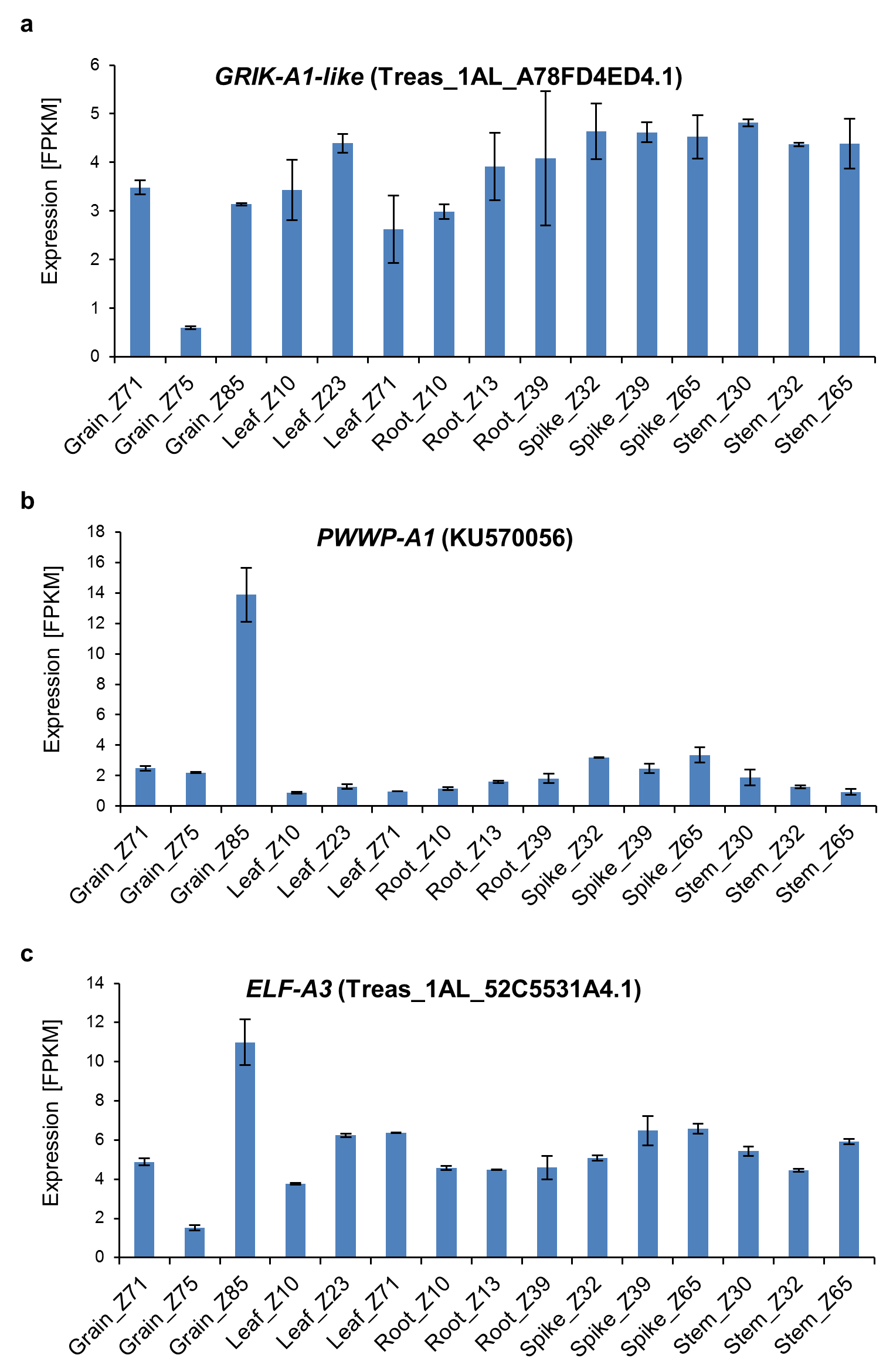
**

**Supplementary Tables**

**Table S1** PCR-based markers developed for the high density genetic map of *Eps-A^m^1* in *Triticum monococcum*. *CAP,* cleavage amplified polymorphism; *INDEL,* insertion/deletion polymorphism

| Locus | Marker type | Primers(5′–3′) | Anneal (°C)^c^ | Restriction enzyme |
| --- | --- | --- | --- | --- |
| *wg241*^a^ | CAP | CTGCATGACTGTCAACTACGC | 55 | *Rsa*I |
|  |  | CTCACCATATGCTGCTGACC |  |  |
| *CA608558*^a^ | CAP | GGATGTTCCCCTTCCTCCT | 55 | *Tse*I |
|  |  | TTCACCATTCATCCGTCTCA |  |  |
| *VATPC*^a^ | CAP | AGGTTAGAGTTGCTGAATACGG | 55 | *Bsm*BI |
|  |  | CAAACGTATCAAGTGACTCGTAGC |  |  |
| *CF2*^b^ | CAP | TTTTGGGCTACTCGACAACC | 55 | *Xba*I |
|  |  | TCTAAACAAGCAAGGGGACCT |  |  |
| *UCW142* | CAP | GATGCGGGGTTGTCTTACAG | 55 | *Sml*I |
|  |  | GGATGGATTTCGCATTCATT |  |  |
| *FOP1* | CAP | TTGGTTCTTTTAGAATTTATTCACG | TD 55-50^d^ | *Eco*P15I |
|  |  | CCCTTGGAAACATGGTAGCC |  |  |
| *MOT1* | indel | ATCCCCAGGAGCTAAATGCT | 55 | - |
|  |  | CAAAACCATGCATTCCTTCA |  |  |
| *FTSH4* | indel | GCGCCCTAATAAACCCTAGC | 55 | - |
|  |  | CTCCGCGAACAAGATTCCT |  |  |
| *ADK1* | indel | TGTTGAATTAATGGTCTACCTATCACA | 55 | - |
|  |  | GGGGCATATGTTCACTAGAGG |  |  |
| *CK1-like* | CAP | CCTACGCGGTTCAGGAGACT | TD 60-54^d^ | *Tsp*45I |
|  |  | ACCACCGGACATTTGGAAT |  |  |
| *ELF3* | CAP | GATGTACTTCCCGCCTTTCA | TD 63-58^d^ | *Apa*I |
|  |  | CAGGCACTATAGCTGCACATAC |  |  |
| *GRIK1-like* | indel | CCGTAACATGGAGCCCATTA | 55 | - |
|  |  | TGCGCGTATACTTAGGTTACCA |  |  |
| *PWWP1* | CAP | GGGCCTTGTGTGAGAGAGAG | TD 60-54^d^ | *Bsr*BI |
|  |  | CTCTGAATGCTTTTTGGGGA |  |  |
| *ACA7* | indel | TTAAGCCACTTGCCCTCAAC | 55 | - |
|  |  | TCCACTCCACCAATTATCATCTC |  |  |
| *NLE* | CAP | AGGCTGTATTGGCCGTTTC | 55 | *Rsa*I |
|  |  | CGTAAGAGGAGTGCCCAAT |  |  |
| *SMP*^a^ | indel | GCAAGGGAAGAGAAAAGCAG | 55 | - |
|  |  | TTTCTCAATCTCATGTTATCCTTCA |  |  |
| *NDK3^a^* | indel | CTTGATGGCGATGAAGGTG | 55 | - |
|  |  | ATGCTCCAGGATCAGGAGGT |  |  |

^a^Valarik et al. (2006). ^b^ Faricelli et al. (2010). ^c^ All PCR products were amplified using 1 min extension times. ^d^ An initial touch down cycle was included starting at the higher temperature and decreasing by 0.5°C /cycle to the lower value shown

**Table S2** PCR-based markers used for the screening of the *Triticum monococcum* BAC library

| Marker | Primers (5′–3′) | Anneal (°C) | Size (bp) | BACs yielded |
| --- | --- | --- | --- | --- |
| *ADK1* | CCGCAGCTGCTGTATGAGT | 58 | 1109 | 707L18 |
|  | CATGTCATCCCTCCTGCTTT |  |  |  |
| *707* | GACTGGGAAGCGACCAACTA | 68-63^a^ | 332 | 503O01, 266D12 |
|  | ACTCTCTCGGTCTTCATCTTTGC |  |  |  |
| *503* | ACACTGAAGCACAGAGACTTGG | 65-60^a^ | 1322 | 715L03, 606E22, 583G12 |
|  | TGCACAACCGCTTATCTCAG |  |  |  |
| *194* | GACTAAACTTCCCACCACCTTG | 63-58^a^ | 1477 | 341L19, 10D08 |
|  | TCGTTGAATTGACACGGATT |  |  |  |
| *395* | ATCGATCATGGAGGTGAAGGAA | 58 | 1900 | 194K07 |
|  | TCATTGGCAATACAAAATCATC |  |  |  |
| *ELF3* | GGGATCGACAGACCTCTCTTT | 62 | 952 | 395E07, 512G15 |
|  | CGATAGCTCTTCTTGCTTTCC |  |  |  |
| *512* | GAAATACATGACATGCAAGCAAT | 68-62^a^ | 365 | 641A14, 710J19, 714J21 |
|  | GCCAAGTTACACTTGAGCAAGAA |  |  |  |
| *714* | GGCATATTTCCAACAGGTTCC | 60-55^a^ | 956 | 220M04 |
|  | TTAGAGGTTGCTTGGCAGGT |  |  |  |
| *SMP* | GCAAGGGAAGAGAAAAGCAG | 55 | 169 | 102H21, 73J03 |
|  | TTTCTCAATCTCATGTTATCCTTCA |  |  |  |

^a^ An initial touch down cycle was included starting at the higher temperature and decreasing by 0.5°C /cycle to the lower value shown

**Table S3** GenBank accession numbers of BAC and gene sequences deposited in this work

| GenBank ID Number | Genetic Background | BAC | Gene(s) |
| --- | --- | --- | --- |
| AC270223.1 | DV92 | 707L18 | *MOT1, FTSH4, ADK1* |
| AC270211.1 | DV92 | 503O01 | *-* |
| AC270216.1 | DV92 | 266D12 | *-* |
| AC270215.1 | DV92 | 715L03 | *CK1-like* |
| AC270204.1 | DV92 | 606E22 | *CK1-like* |
| AC270213.1 | DV92 | 583G12 | *CK1-like* |
| AC270219.1 | DV92 | 341L19 | *-* |
| AC270212.1 | DV92 | 10D08 | *-* |
| AC270214.1 | DV92 | 194K07 | *-* |
| AC270217.1 | DV92 | 395E07 | *ELF3, GRIK1-like* |
| AC270218.1 | DV92 | 512G15 | *ELF3, GRIK1-like* |
| AC270208.1 | DV92 | 641A14 | *-* |
| AC270203.1 | DV92 | 710J19 | *PWWP1* |
| AC270207.1 | DV92 | 714J21 | *PWWP1* |
| AC270206.1 | DV92 | 220M04 | *-* |
| AC270205.1 | DV92 | 102H21 | *ACA7, NLE, SMP* |
| AC270209.1 | DV92 | 73J03 | *ACA7, NLE, SMP* |
| KU570055 | G3116 | - | *ELF3* |
| KU570056 | G3116 | - | *PWWP1* |
| KU570057 | G3116 | - | *GRIK1-like* |

**Table S4** PCR primers and conditions used for the amplification of candidate genes *ELF3*, *GRIK1-like,* and *PWWP1* in both parents of the *T. monococcum* mapping population

| Gene | Target | Primers (5′–3′) | Anneal (°C) | Size (bp) |
| --- | --- | --- | --- | --- |
| *ELF3* | Exon 1 | TGATGGCGTCCTAGCTAGTTG | TD 63-58^a^ | 1100 |
|  |  | AAAGCCTCCCAGATGTAGCA |  |  |
|  | Exon 2 | GGGATCGACAGACCTCTCTTT | 62 | 952 |
|  |  | CGATAGCTCTTCTTGCTTTCC |  |  |
|  | Exon 3 | CAGATGATGTTGTCGGTGCT | 64 | 686 |
|  |  | TTGATCAGTCGATGCAGCTC |  |  |
|  | Exon 4 | GGGTTTTTGCTGTCCAAGTG | 64 | 790 |
|  |  | AGACCACCGGGTTCATCACT |  |  |
|  | Exon 4 | GATGTACTTCCCGCCTTTCA | TD 63-58^a^ | 866 |
|  |  | CAGGCACTATAGCTGCACATAC |  |  |
| *GRIK1-like* | Exon 1 | GTTGCCGAGGATTTTGAGAG | 59 | 699 |
|  |  | GGAAGAGCTTCCTGGTGTCC |  |  |
|  | Exon 2 | GACGGCGGCAGCTTCTAC | 59 | 1182 |
|  |  | CCGTCCTTGATGTTTCGGTA |  |  |
|  | Exons 3, 4, 5 | AAGATTGGTGCTGGGAGCTA | 59 | 1128 |
|  |  | AAACCTATGCCGTTATTGCAC |  |  |
|  | Exons 6, 7 | AGGTGATTGACGACCCAAAC | 59 | 1309 |
|  |  | CCTCAAATATCTGGCTAACACTGA |  |  |
|  | Exons 8, 9, 10 | TTCATGGTGATATTAAACCAGACAA | 59 | 1230 |
|  |  | CCGCACCTACACACAAACTC |  |  |
|  | Exon 11 | CCGGGAAATATCCATTCCTT | TD 60-54^a^ | 756 |
|  |  | TTCAAAATTCACCCATGGAA |  |  |
| *PWWP1* | Exon 1 | AGGGAGATCGGTCGAATTG | 72^b^ | 641 |
|  |  | GATCCGATGTCGGTGAGGT |  |  |
|  | Exon 2 | AGGAGCAAGCGCGTGAAG | 72^b^ | 621 |
|  |  | AGGTTGATCCACAGGACCAC |  |  |
|  | Exons 2, 3 | AGCAATGGCTTGGATACGTC | TD 70-65^a^ | 1377 |
|  |  | CTTCAGGCTCGCACTTGATT |  |  |
|  | Exon 3 | ATTTGCACGCCCACCAAC | TD 70-65^a^ | 881 |
|  |  | ATCCCGTACCCGACCAAC |  |  |
|  | Exon 3 | TCCCTGTACGACGTGGAAAT | TD 70-65^a^ | 1062 |
|  |  | TTAGAGCAGACACACAGAAACAG |  |  |

^a^ An initial touch down cycle was included starting at the higher temperature and decreasing by 0.5°C /cycle to the lower value shown. ^b^ Two step program using Phusion^®^ High-Fidelity DNA Polymerase and GC buffer

**Table S5** Primers used for qRT-PCR analysis of candidate genes, flowering genes, and circadian clock genes

| Gene | Primers (5′–3′) | Reference |
| --- | --- | --- |
| *ACTIN* | ACCTTCAGTTGCCCAGCAAT | Distelfeld et al. (2009) |
|  | CAGAGTCGAGCACAATACCAGTTG |  |
| *CCA1* | CCTGGAATTGGAGATGGAGA | Campoli et al. (2012) |
|  | TGAGCATGGCTTCTGATTTG |  |
| *ELF3* | CTGTCCAAGTGTTCGAGCTG |  |
|  | GCCGTCTTCTTCTTGCTTGT |  |
| *FT1* | CAGCAGCCCAGGGTTGAG | Yan et al. (2006) |
|  | ATCTGGGTCTACCATCACGAGTG |  |
| *GIGANTEA* | TCTGGATTGCTCGAGATGAC | Shaw et al. (2012) |
|  | AAGCTTCACCGTCGACAA |  |
| *GRIK1-like* | GCCAGATATTTGAGGATGACG |  |
|  | ATGGTAGGCTGAACCTTGACA |  |
| *PIF-like* | AGAGGAGGAGAAGGGACAGG |  |
|  | GTTGCAGTGGGGTACGAGTT |  |
| *PPD1* | CGGCATTCACGAGGTACAATAC | Chen et al. (2014) |
|  | GAGCCTTGCTTCATCTGAGCG |  |
| *PRR59* | GCGTAACTTATGGCAACAT | Chen et al. (2014) |
|  | CTGAGCATCACTTTCCTC |  |
| *PRR73* | TGGAACTGAAATCCATGATGA | Shaw et al. (2012) |
|  | TGGGAGTATGGTACCTGGTG |  |
| *PRR95* | CAACGTGAGATTGCACTGAA | Chen et al. (2014) |
|  | CTCTGGTACCGCACCTTCTT |  |
| *PWWP1* | GACTATCTTGAAAGCCGATCAT |  |
|  | CTTGGTGCATTCCCAGGTAG |  |
| *TOC1* | CCAAAACAGACCAATTGGAAA | Shaw et al. (2012) |
|  | TCCTCCTGGAGATGATGCTT |  |

**Table S6** Genome-specific primers and PCR conditions used for the detection of TILLING mutations in gene *ELF3*

| Gene | Target | Primers (5′–3′) | Anneal (°C) | Size (bp) |
| --- | --- | --- | --- | --- |
| *ELF3-A* | Exons 3, 4 | TGTAATCACTTTTCTTTGAACTCTCTA | TD 58-52^a^ | 1329 |
|  |  | CAAAAATTCGCTTTTACCGTCA |  |  |
| *ELF3-B* | Exons 3, 4 | ATCACTATAACTTCCTTTCTTGTGTA | TD 58-54^a^ | 1421 |
|  |  | CAAAAATTCGCTTTTACCGATG |  |  |

^a^ An initial touch down cycle was included starting at the higher temperature and decreasing by 0.5°C /cycle to the lower value shown

**Table S7** Molecular markers mapped to chromosome 1A, polymorphic between *T. monococcum* accession DV92 and *T. turgidum* ssp. *durum* cultivar CBW0112. *CAP,* cleavage amplified polymorphism; *SSR,* simple sequence repeats. All PCR products were amplified using 1 min extension time

| Locus | Marker Type | Chromosome Arm location | Primers (5′–3′) | Anneal (°C) | Restriction enzyme |
| --- | --- | --- | --- | --- | --- |
| *GLU-A3* | SSR | 1AS | TCCCGCCATGAGTCAATC | TD 60-55 | - |
|  |  |  | TTGGGAGACACATTGGCC |  |  |
| *barc263* | SSR | 1AS | GGAAGCGCGTCAGCACTAGGCAAC | 55 | - |
|  |  |  | GGCTTCTAGGTGCTGCGGCTTTTGTC |  |  |
| *gwm136* | SSR | 1AS | GACAGCACCTTGCCCTTTG | 60 | - |
|  |  |  | CATCGGCAACATGCTCATC |  |  |
| *gwm164* | SSR | 1AL | ACATTTCTCCCCCATCGTC | 55 | - |
|  |  |  | TTGTAAACAAATCGCATGCG |  |  |
| *gwm135* | SSR | 1AL | TGTCAACATCGTTTTGAAAAGG | 60 | - |
|  |  |  | ACACTGTCAACCTGGCAATG |  |  |
| *gwm357* | SSR | 1AL | TATGGTCAAAGTTGGACCTCG | 55 | - |
|  |  |  | AGGCTGCAGCTCTTCTTCAG |  |  |
| *wmc312* | SSR | 1AL | TGTGCCCGCTGGTGCGAAG | 61 | - |
|  |  |  | CCGACGCAGGTGAGCGAAG |  |  |
| *wmc716* | SSR | 1AL | CATTTATGTGCACGCCGAAG | 61 | - |
|  |  |  | CCATAAGCATCGTCACCCTG |  |  |
| *wg241* | CAP | 1AL | CTGCATGACTGTCAACTACGC | 55 | *Rsa*I |
|  |  |  | CTCACCATATGCTGCTGACC |  |  |
| *ELF3* | CAP | 1AL | GAAGAGAGGACTTCATCATTTCA | TD 60-55 | *Pst*I |
|  |  |  | TGCCTGGCTCATTTGCTTA |  |  |
| *NDK3* | CAP | 1AL | CTTGATGGCGATGAAGGTG | 55 | - |
|  |  |  | ATGCTCCAGGATCAGGAGGT |  |  |
| *barc287* | SSR | 1AL | CGGATGGGTTACTTACTTAGGATG | 50 | - |
|  |  |  | CGCAACTCCATTTCAGAATCAT |  |  |

^a^ An initial touch down cycle was included starting at the higher temperature and decreasing by 0.5°C /cycle to the lower value shown

**Table S8** Haplotypes of candidate proteins

|  |  |  | **ELF3** | **ELF 3 Protein** | | | |  | **GRIK1-like Protein** | | |  | **PWWP1 Protein** | | | | |
| --- | --- | --- | --- | --- | --- | --- | --- | --- | --- | --- | --- | --- | --- | --- | --- | --- | --- |
| **Genotype** | **Habit** | ***Eps1^a^*** | **Hap.** | V364L^b^ | G681R | G700D | G718A |  | D77E | A228S | D351N |  | S251W | T294M | S327I | G542C | V701A |
| *T. urartu* (PI 428198) | Spring |  | A | V | G | G | G |  | D | A | D |  | S | T | S | G | V |
| *T. turgidum* ssp. *durum*-A (Kronos) | Spring |  | A | V | G | G | G |  | D | A | D |  | S | T | S | G | V |
| *T. turgidum* ssp. *durum*-B (Kronos) | Spring |  | A | V | G | G | G |  | D | A | D |  | T | T | S | G | V |
| *T. aestivum*-D (CS) | Spring |  | A _(V364A)_ | A | G | G | G |  | D | A | D |  | -- | -- | -- | -- | -- |
| *Hordeum vulgare* (Bowman) | Spring |  | A _(G681A)_ | V | A | G | G |  | D | A | D |  | S | P | S | G | V |
| *Brachypodium distachyon* (Bd21) | Spring |  | A _(G681W)_ | V | W | G | G |  | D | A | D |  | L | G | S | -- | -- |
| *Sorghum bicolor* (BTx623) | Spring |  | A _(G718R)_ | V | G | G | R |  | D | A | D |  | P | Q | R | -- | -- |
| *T. monococcum* ssp. *monococcum* (PI 355517) | Spring |  | A | V | G | G | G |  | D | A | D |  | S | M | I | C | V |
| *T. monococcum* ssp. *monococcum* (PI 573525) | Winter |  | A | V | G | G | G |  | D | A | D |  | S | M | I | C | V |
| *T. monococcum* ssp. *monococcum* (PI 560729) | Winter |  | A | V | G | G | G |  | D | A | D |  | S | M | S | G | A |
| *T. monococcum* ssp. *aegilopides* (G3116) | Winter | early | B | V | G | D | A |  | E | S | N |  | W | T | I | C | V |
| *T. monococcum* ssp. *aegilopides* (G2528) | Spring |  | B | V | G | D | A |  | E | S | D |  | W | M | I | C | V |
| *T. monococcum* ssp. *aegilopides* (G1777) | Winter |  | B | V | G | D | A |  | E | S | D |  | W | M | I | C | V |
| *T. monococcum* ssp. *monococcum* (PI 596286) | Winter | early | B | V | G | D | A |  | E | S | D |  | W | M | I | C | V |
| *T. monococcum* ssp. *monococcum* (PI 573529) | Winter | early | B | V | G | D | A |  | E | S | D |  | W | M | I | C | V |
| *T. monococcum* ssp. *monococcum* (PI 355522) | Winter | early | B | V | G | D | A |  | E | S | N |  | S | M | I | C | V |
| *T. monococcum* ssp. *monococcum* (PI 355515) | Winter |  | B | V | G | D | A |  | E | S | D |  | S | M | I | C | V |
| *T. monococcum* ssp. *monococcum* (PI 272557) | Spring |  | B | V | G | D | A |  | E | S | D |  | S | M | I | C | V |
| *T. monococcum* ssp. *monococcum* (DV92) | Spring | late | C | L | R | G | G |  | D | A | D |  | S | M | S | G | A |
| *T. monococcum* ssp. *monococcum* (PI 352484) | Spring |  | C | L | R | G | G |  | D | A | D |  | S | M | S | G | A |
| *T. monococcum* ssp. *monococcum* (PI 345242) | Spring |  | C | L | R | G | G |  | D | A | D |  | S | M | S | G | A |
| *T. monococcum* ssp. *monococcum* (PI 277130) | Winter |  | C | L | R | G | G |  | D | A | D |  | S | M | S | G | A |
| *T. monococcum* ssp. *monococcum* (PI 272561) | Winter |  | C | L | R | G | G |  | D | A | D |  | S | M | S | G | A |
| *T. monococcum* ssp. *monococcum* (PI 326317) | Spring | late | C | L | R | G | G |  | D | A | D |  | S | M | S | G | A |
| *T. monococcum* ssp. *monococcum* (PI 306540) | Spring | late | C | L | R | G | G |  | D | A | D |  | S | M | S | G | A |
| *T. monococcum* ssp. *monococcum* (PI 591871) | Spring |  | C | L | R | G | G |  | D | A | D |  | S | M | S | G | A |
| *T. monococcum* ssp. *monococcum* (PI 503874 ) | Spring | late | C | L | R | G | G |  | D | A | D |  | S | M | S | G | A |
| *T. monococcum* ssp. *monococcum* (PI 428175) | Spring |  | C | L | R | G | G |  | D | A | D |  | S | M | S | G | A |
| *T. monococcum* ssp. *monococcum* (PI 272560 ) | Spring |  | C | L | R | G | G |  | D | A | D |  | S | M | S | G | A |
| *T. monococcum* ssp. *monococcum* (PI 266844) | Spring | late | C | L | R | G | G |  | D | A | D |  | S | M | S | G | A |
| *T. monococcum* ssp. *monococcum* (PI 221415) | Spring |  | C | L | R | G | G |  | D | A | D |  | S | M | S | G | A |
| *T. monococcum* ssp. *monococcum* (PI 428166) | Spring |  | C | L | R | G | G |  | D | A | D |  | S | M | S | G | A |
| *T. monococcum* ssp. *monococcum* (PI 428154) | Spring |  | C | L | R | G | G |  | D | A | D |  | S | M | S | G | A |
| *T. monococcum* ssp. *monococcum* (PI 428152) | Spring |  | C | L | R | G | G |  | D | A | D |  | S | M | S | G | A |
| *T. monococcum* ssp. *monococcum* (PI 428151) | Spring |  | C | L | R | G | G |  | D | A | D |  | S | M | S | G | A |
| *T. monococcum* ssp. *monococcum* (PI 428149) | Spring |  | C | L | R | G | G |  | D | A | D |  | S | M | S | G | A |
| *T. monococcum* ssp. *monococcum* (PI 427927) | Spring |  | C | L | R | G | G |  | D | A | D |  | S | M | S | G | A |
| *T. monococcum* ssp. *monococcum* (PI 393496) | Spring |  | C | L | R | G | G |  | D | A | D |  | S | M | S | G | A |
| *T. monococcum* ssp. *monococcum* (PI 427959) | Spring |  | C | L | R | G | G |  | D | A | D |  | S | M | S | G | A |
| *T. monococcum* ssp. *monococcum* (PI 377648) | Spring |  | C | L | R | G | G |  | D | A | D |  | S | M | S | G | A |
| *T. monococcum* ssp. *monococcum* (PI 355546) | Spring |  | C | L | R | G | G |  | D | A | D |  | S | M | S | G | A |
| *T. monococcum* ssp. *monococcum* (PI 355534) | Winter |  | C | L | R | G | G |  | D | A | D |  | S | M | S | G | A |
| *T. monococcum* ssp. *monococcum* (PI 355532) | Winter |  | C | L | R | G | G |  | D | A | D |  | S | M | S | G | A |
| *T. monococcum* ssp. *monococcum* (PI 355523) | Spring |  | C | L | R | G | G |  | D | A | D |  | S | M | S | G | A |
| *T. monococcum* ssp. *monococcum* (PI 355540) | Winter |  | C | L | R | G | G |  | D | A | D |  | S | M | S | G | A |
| *T. monococcum* ssp. *monococcum* (PI 191096) | Spring | late | C | L | R | G | G |  | D | A | D |  | S | M | S | G | A |
| *T. monococcum* ssp. *monococcum* (PI 191383) | Spring |  | C | L | R | G | G |  | D | A | D |  | S | M | S | G | A |
| *T. monococcum* ssp. *monococcum* (PI 10474) | Spring |  | C | L | R | G | G |  | D | A | D |  | S | M | S | G | A |
| *T. monococcum* ssp. *monococcum* (PI 192063) | Spring | late | C | L | R | G | G |  | D | A | D |  | S | M | S | G | A |

^a^ Fully vernalized plants grown in a controlled environment (16h light with fluorescent lamps, see Supplementary Table S9)

^b^ The first letter indicates the *T. urartu* allele, the number indicates the position in the predicted *T. urartu* protein (Supplementary Figures S1-3), and the last letter indicates the derived amino acid

**Table S9**. Characterization of the *Eps-A^m^1* allele from PI 355522^a^

| Eps-Am1 Groups | Count | Avg. Heading | SE |  | Avg. Spikelet No. | SE |
| --- | --- | --- | --- | --- | --- | --- |
| Homozygous DV92 | 6 | 131.0 | 6.0 |  | 27.9 | 1.5 |
| Heterozygous | 19 | 100.4 | 2.1 |  | 24.2 | 0.7 |
| Homozygous PI 355522 | 10 | 83.4 | 1.8 |  | 20.7 | 1.3 |
| *P* value ANOVA |  | 3.711E-10 |  |  | 0.0013 |  |

^a^ *T. monococcum* accession PI 355522 (ELF3 haplotype-B, Supplementary Table S8) was crossed with DV92 (ELF3 haplotype-C) and the F_1_ was backcrossed to DV92. A BC_1_ line homozygous for the DV92 *vrn-A^m^2* allele for spring growth habit and heterozygous for *Eps-A^m^1* was selected to generate a population segregating for *Eps-A^m^1* in a spring background. BC_1_F_2_ plants were grown under controlled environmental conditions (16 °C constant temperature, 16 hours fluorescent lamps, 160 µM m^–2^ s^–1^ fluency) and heading time, from sowing to ear emergence, and number of spikelets per spike were registered

**Table S10**. Characterization of *T. monococcum* accessions carrying the ELF3 haplotypes B and C^1^

| Genotype | Habit | ELF3 haplotype | Heading time | Tukey test |
| --- | --- | --- | --- | --- |
| *T. monococcum* ssp. *aegilopides* (G3116) | Winter | B | 63.5 ± 1.0 | a |
| *T. monococcum* ssp. *aegilopides* (G2528) | Spring | B | 72.3 ± 1.9 | a |
| *T. monococcum* ssp. *monococcum* (PI 596286)^2^ | Winter | B | 70.8 ± 1.5 | a |
| *T. monococcum* ssp. *monococcum* (PI 573529) ^2^ | Winter | B | 76.0 ± 2.5 | a |
| *T. monococcum* ssp. *monococcum* (PI 355522) ^2^ | Winter | B | 73.7 ± 2.5 | a |
| *T. monococcum* ssp. *monococcum* (DV92) | Spring | C | 109.4 ± 5.9 | b |
| *T. monococcum* ssp. *monococcum* (PI 326317) | Spring | C | 119.0 ± 9.8 | b |
| *T. monococcum* ssp. *monococcum* (PI 306540) | Spring | C | 108.8 ± 2.7 | b |
| *T. monococcum* ssp. *monococcum* (PI 503874 ) | Spring | C | 115.7 ± 4.8 | b |
| *T. monococcum* ssp. *monococcum* (PI 266844) | Spring | C | 114.7 ± 3.4 | b |
| *T. monococcum* ssp. *monococcum* (PI 191096) | Spring | C | 113.8 ± 3.0 | b |
| *T. monococcum* ssp. *monococcum* (PI 192063) | Spring | C | 106.7 ± 3.1 | b |

^1^Plants were vernalized for 6 weeks at 4 °C and then were grown under controlled environmental conditions (16 °C constant temperature, 16 hours fluorescent lamps, 160 µM m^–2^ s^–1^ fluency). Average heading time is expressed in days from sowing to ear emergence ± standard error of the mean. Lines with the same letter in the Tukey test are not significantly different from each other. The two haplotypes were well separated in two groups.

^2^Lines evaluated in the same chamber and under the same conditions but in a separate experiment. DV92 was used as a common control.

**Table S11**. Epistatic interactions between *ELF3* and *PPD1*

***Spikelet number wheat ****

|  | LD | | SD | |
| --- | --- | --- | --- | --- |
|  | Spikelet No. | *P* | Spikelet No. | *P* |
| *ELF3* effect in *PPD-A1a* | **3.8** | <0.0001 | **6.5** | <0.0001 |
| *ELF3* effect in *PPD-A1b* | **6.4** | <0.0001 | **11.6** | <0.0001 |
|  |  |  |  |  |
| *PPD-A1* effect in *ELF3* | 1.4 | 0.2062 | **6.0** | 0.0156 |
| *PPD-A1* effect in *elf3* | **-1.2** | 0.0250 | 0.9 | 0.1236 |

*Interactions *ELF3* x *PPD1* LD *P*= 0.0164, SD *P=*0.223

***Heading time wheat****

|  | LD | | SD | |
| --- | --- | --- | --- | --- |
|  | Heading time (d) | *P* | Heading time (d) | *P* |
| *ELF3* effect in *PPD-A1a* | **7.2** | 0.0062 | **26.6** | <0.0001 |
| *ELF3* effect in *PPD-A1b* | **23.2** | <0.0001 | **91.4** | <0.0001 |
|  |  |  |  |  |
| *PPD-A1* effect in *ELF3* | **14.0** | <0.0001 | **66.3** | <0.0001 |
| *PPD-A1* effect in *elf3* | -2.0 | 0.3943 | 1.4 | 0.05092 |

*Interactions *ELF3* x *PPD1* LD= *P<*0.0001, SD= *P<*0.0001

***Heading time barley**** (Faure et al. 2012, Fig. 1)

|  | LD | | SD | |
| --- | --- | --- | --- | --- |
|  | Heading time (d) | *P* | Heading time (d) | *P* |
| *ELF3* effect in *ppd-H1* | **13.3** | * | **25.3** | * |
| *ELF3* effect in *PPD-H1* | **2.2** | * | **24.7** | * |
|  |  |  |  |  |
| *PPD-H1* effect in *ELF3* | **18.5** | * | **0.5** | NS |
| *PPD-A1* effect in *elf3* | **7.3** | * | **0.2** | NS |

*Interactions *ELF3* x *PPD-H1* LD= *, SD = NS

**References**

Campoli C, Shtaya M, Davis SJ, von Korff M (2012) Expression conservation within the circadian clock of a monocot: natural variation at barley *Ppd-H1* affects circadian expression of flowering time genes, but not clock orthologs. BMC Plant Biol 12:1

Chen A, Li C, Hu W, Lau ML, Lin H, Rockwell NC, Martin SS, Jernstedt JA, Lagarias JC, Dubcovsky J (2014) *PHYTOCHROME C* plays a major role in the acceleration of wheat flowering under long day photoperiod. Proc Natl Acad Sci USA 111: 10037–10044

Distelfeld A, Tranquilli G, Li C, Yan L, Dubcovsky J (2009) Genetic and molecular characterization of the *VRN2* loci in tetraploid wheat. Plant Physiol 149:245–257

IWGSC The International Wheat Genome Sequencing Consortium (2014) A chromosome-based draft sequence of hexaploid bread wheat (*Triticum aestivum*) genome. Science 345:1251788

Pearce S, Vazquez-Gross H, Herin SY, Hane D, Wang Y, Gu YQ, Dubcovsky J (2015) WheatExp: an RNA-seq expression database for polyploid wheat. BMC Plant Biol 15:299

Shaw LM, Turner AS, Laurie DA **The impact of photoperiod insensitive *Ppd-1a* mutations on the photoperiod pathway across the three genomes of hexaploid wheat (*Triticum* aestivum).** Plant J*.* **71**:71–84

Somers DJ, Isaac P, Edwards K (2004) A high-density microsatellite consensus map for bread wheat (*Triticum aestivum* L.). Theor Appl Genet 109:1105–14

Yan L, Fu D, Li C, Blechl A, Tranquilli G, Bonafede M, Sanchez A, Valarik M, Yasuda S, Dubcovsky J. (2006) The wheat and barley vernalization gene *VRN3* is an ortholog of *FT*. Proc Natl Acad Sci USA 103: 19581–19586
